# The androgen-induced protein AIbZIP facilitates proliferation of prostate cancer cells through downregulation of p21 expression

**DOI:** 10.1038/srep37310

**Published:** 2016-11-17

**Authors:** Xiang Cui, Min Cui, Rie Asada, Soshi Kanemoto, Atsushi Saito, Koji Matsuhisa, Masayuki Kaneko, Kazunori Imaizumi

**Affiliations:** 1 Department of Biochemistry, Institute of Biomedical and Health Sciences, Hiroshima University, Hiroshima 734-8553, Japan

## Abstract

Androgen-Induced bZIP (AIbZIP) is structurally a bZIP transmembrane transcription factor belonging to the CREB/ATF family. This molecule is highly expressed in androgen-sensitive prostate cancer cells and is transcriptionally upregulated by androgen treatment. Here, we investigated molecular mechanism of androgen-dependent expression of AIbZIP and its physiological function in prostate cancer cells. Our data showed that SAM pointed domain-containing ETS transcription factor (SPDEF), which is upregulated by androgen treatment, directly activates transcription of *AIbZIP*. Knockdown of *AIbZIP* caused a significant reduction in the proliferation of androgen-sensitive prostate cancer cells with robust expression of *p21*. Mechanistically, we demonstrated that AIbZIP interacts with old astrocyte specifically induced substance (OASIS), which is a CREB/ATF family transcription factor, and prevents OASIS from promoting transcription of its target gene *p21*. These findings showed that AIbZIP induced by the androgen receptor (AR) axis plays a crucial role in the proliferation of androgen-sensitive prostate cancer cells, and could be a novel target of therapy for prostate cancer.

The growth and survival of prostate cancer depend on androgens at an early stage^1^,^2^. Testosterone, which is the main circulating androgen, is produced mainly in the testes and circulates in the blood^1^. When testosterone is incorporated into the cells of the prostate, it is converted to dihydrotestosterone (DHT)^1^, which binds to the androgen receptor (AR), a member of the steroid-thyroid-retinoid nuclear receptor superfamily^1^. The AR then translocates to the nucleus, dimerizes, and binds to the androgen response element (ARE) in the promoter regions of target genes such as *prostate-specific antigen (PSA*)^1^,^3^. The downstream genes of the AR axis function in the development, growth, and survival of normal prostate tissue as well as prostate cancer^4^. However, little is known about which AR target genes are essential for the proliferation of prostate cancer cells.

Hormone therapy or androgen deprivation therapy (ADT) that reduce androgen levels or block androgen activity are administered to inhibit the growth of prostate cancer at the initial stage^2^. However, the majority of prostate cancers eventually develop into castration-resistance prostate cancer (CRPC)^2^, which means that the cancer cells are still able to proliferate despite ADT. Therefore, understanding the physiological functions and the regulatory mechanisms responsible for the expression of AR target genes is absolutely critical for both elucidation of pathogenic mechanisms and the development of therapeutic strategies for prostate cancer.

Some endoplasmic reticulum (ER)- resident CREB/ATF family members are involved in the unfolded protein response, which is a response system against ER stress caused by the accumulation of unfolded proteins in the ER^5^,^6^. These include the OASIS family members; Luman^7^, OASIS^8^, BBF2H7^9^, CREBH^10^, and AIbZIP^11^, which share sequence similarities with the representative ER stress sensor ATF6^12^. OASIS family proteins are commonly translocated from the ER to the Golgi apparatus in response to various stimuli including ER stress, and sequentially cleaved by site-1 protease (S1P) and site-2 protease (S2P)^13^,^14^. Cleaved N-termini containing transcription activation and basic leucine zipper (bZIP) domains then translocate to the nucleus to promote gene expression^14^,^15^. It has been demonstrated that the OASIS family members have cell- or tissue-specific distribution and are involved in defined physiological functions including differentiation and proliferation^16^.

*AIbZIP* was originally identified as a gene that is upregulated in androgen-treated prostate cancer cell lines^11^. Previous studies reported that AIbZIP is highly expressed in the human prostate and is more abundant in prostate cancer compared with normal prostate tissue^11^,^17^,^18^. Moreover, the expression levels of AIbZIP were shown to be increased in malignant tissue compared with benign tissue^17^,^18^, suggesting that AIbZIP might contribute to the development and/or progression of prostate cancer induced by AR signaling. The mouse homologue of AIbZIP, Tisp40^19^ or ATCE1^20^, has also been identified. Unlike human AIbZIP, mouse AIbZIP is specifically expressed in testis^20^,^21^. Although human and mouse AIbZIP show high structural similarity^21^, their expression patterns display different tissue distributions^11^,^20^, indicating limitations to the use of AIbZIP-deficient mice for determining the physiological function of human AIbZIP. Therefore, the roles of human AIbZIP remain incompletely understood. In this study, we investigated the regulatory mechanism of androgen-dependent AIbZIP expression and elucidated its roles in the proliferation of prostate cancer cells.

## Results

### Expression of AIbZIP in androgen-sensitive prostate cancer cell line LNCaP

**Figure 1:**
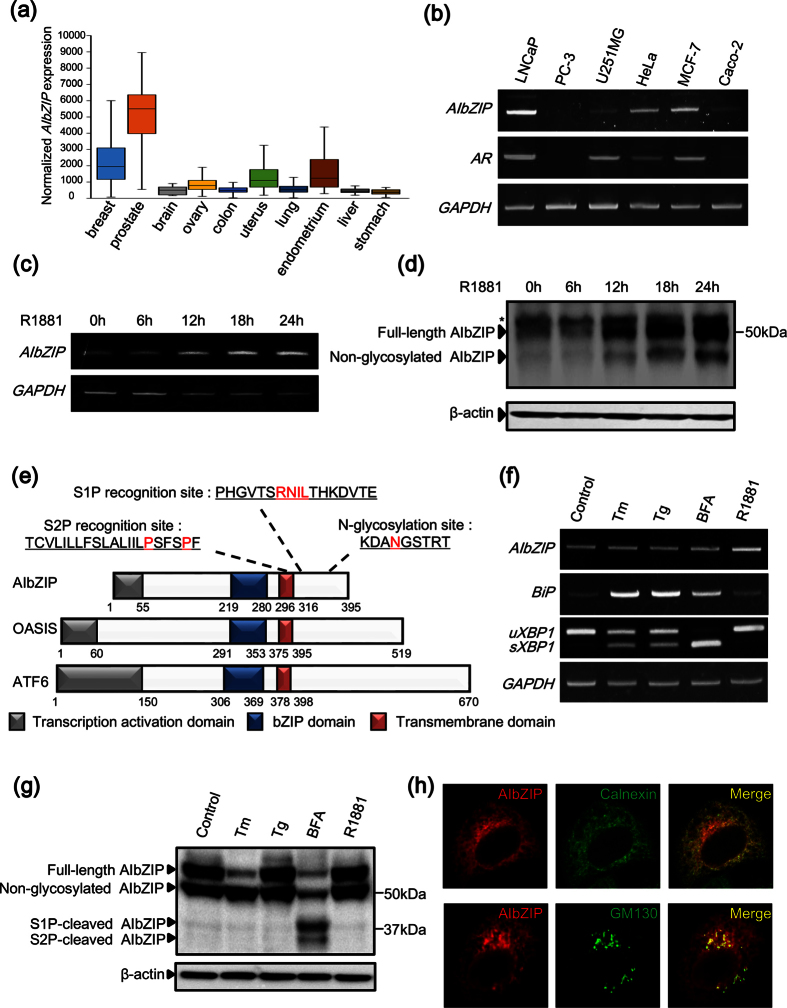
Characterization of AIbZIP in androgen-sensitive prostate cancer cell line LNCaP. (**a**) Microarray datasets were accessed in the ONCOMINE Cancer Profiling Database (version 4.5, www.oncomine.org). The number of each tumor sample is described in Methods section. The y-axis represents the expression levels of normalized *AIbZIP*. The line within the box represents the median expression value for each group, and the upper and lower edges of the box indicate the 75% and 25% limits of distribution, respectively. The lines extending from each box (whiskers) indicate the 90% and 10% limits of distribution. (**b**) RT-PCR analysis for *AIbZIP* and *AR* in indicated cancer cell lines. *GAPDH* was used as an internal control. (**c**) RT-PCR analysis for *AIbZIP* in LNCaP cells treated with R1881 for indicated time periods. (**d**) Western blot (WB) analysis for endogenous AIbZIP in LNCaP cells treated with R1881 as in (**c**). Asterisk: nonspecific bands. β-actin was used as a loading control. (**e**) Schematic representation of the domain structures of human AIbZIP, OASIS, and ATF6. Amino acids colored in red indicate the S1P recognition site, the putative S2P recognition site, and the N-glycosylation site. (**f**) RT-PCR analysis for *AIbZIP* and ER stress markers in LNCaP cells treated with various kinds of ER stressors for 6 h, or R1881 for 24 h. *uXBP1*: unspliced forms of *XBP1*, *sXBP1*: spliced forms of *XBP1*, Tm: tunicamycin, Tg: thapsigargin, BFA: brefeldin A. (**g**) WB analysis for the processing of AIbZIP protein in LNCaP tet-off cells stably expressing FLAG-tagged AIbZIP treated with ER stressors or R1881 as in (**f**). (**h**) HeLa cells were transfected with a vector expressing FLAG-tagged AIbZIP for 24 h, then co-stained with anti-FLAG (AIbZIP) and anti-calnexin or anti-GM130 antibodies. Full-length gels and blots are presented in Supplementary Figure S8.

AIbZIP has been reported to be highly expressed in androgen-treated prostate cancer cell lines^11^. To confirm the upregulation of AIbZIP in prostate cancer, we examined the expression levels of *AIbZIP* in various tumor types using the ONCOMINE Cancer Profiling Database. *AIbZIP* was highly expressed in sex hormone-related cancers including prostate, breast, endometrium, and uterus cancers (Fig. 1a). Notably, the expression levels of *AIbZIP* were much higher in prostate cancer than in other cancers (Fig. 1a), implying that AIbZIP expression could correlate with progression of prostate cancer. An experimental investigation using various cancer cell lines showed the mRNA levels of *AIbZIP* were extremely high in LNCaP (androgen-sensitive prostate cancer) cells, and moderate in MCF-7 (breast cancer) and HeLa (cervical cancer) cells (Fig. 1b). In contrast, *AIbZIP* was hardly detected in PC-3 (androgen-insensitive prostate cancer) and Caco-2 (colon carcinoma) cells (Fig. 1b). Interestingly, cells expressing *AIbZIP* have a tendency to express *AR* (Fig. 1b). These findings suggested a link between AIbZIP expression and AR signaling.

Next, we checked the induction of AIbZIP in response to androgen stimulation. Treatment of LNCaP cells with the synthetic AR agonist R1881 resulted in a time-dependent increase of *AIbZIP* expression at both the mRNA and protein levels (Fig. 1c,d). Interestingly, western blot (WB) analysis showed that two bands of AIbZIP protein at approximately 50 and 43 kDa were increased after treatment with R1881 (Fig. 1d). AIbZIP contains a putative N-glycosylation site within the luminal domain (Fig. 1e). Treatment of cells with tunicamycin (Tm), which blocks N-linked glycosylation, decreased the 50 kDa band and increased the 43 kDa one, indicating that the 50 kDa AIbZIP is a glycosylated form, while the 43 kDa one is not (Supplementary Figure S1).

AIbZIP belongs structurally to the CREB/ATF transcription factor family with a similarity to the ER stress transducers ATF6 and OASIS (Fig. 1e). To determine if AIbZIP is upregulated or activated in response to ER stress, we treated LNCaP cells with ER stressors Tm, thapsigargin (Tg), and brefeldin A (BFA), respectively. The expression of *AIbZIP* mRNA was never changed although ER stress markers *BiP* and spliced *XBP1 (sXBP1*) were upregulated (Fig. 1f). AIbZIP has potential cleavage sites for both S1P and S2P (Fig. 1e). Indeed, treatment with BFA, which is a compound that causes Golgi tubules to fuse with the ER^22^, induced cleavage of AIbZIP (Fig. 1g). However, AIbZIP was never cleaved by ER stressors Tm, Tg, or R1881 (Fig. 1g), indicating that AIbZIP is not activated in response to ER stress and could function as a full-length form in prostate cancer cells. The reason why AIbZIP transported from the ER to the Golgi apparatus is not cleaved by S1P and S2P remains unknown, but it is possible that some molecules inhibit cleavage by masking the recognition sites for S1P and S2P at the Golgi apparatus.

To investigate the subcellular localization of AIbZIP, we performed immunofluorescence staining of HeLa cells transfected with a vector expressing FLAG-tagged AIbZIP (FLAG-AIbZIP). FLAG-AIbZIP immunoreactivity was detected in the perinuclear region (Fig. 1h). Co-staining for FLAG and the ER marker calnexin showed that AIbZIP partially co-localized with calnexin (Fig. 1h). Furthermore, FLAG-AIbZIP signals were also detected from GM130-positive Golgi apparatus (Fig. 1h). These findings implied that AIbZIP could localize and function not only in the ER, but also in the Golgi apparatus as a full-length form.

### AIbZIP is induced by SPDEF acting downstream of AR

**Figure 2:**
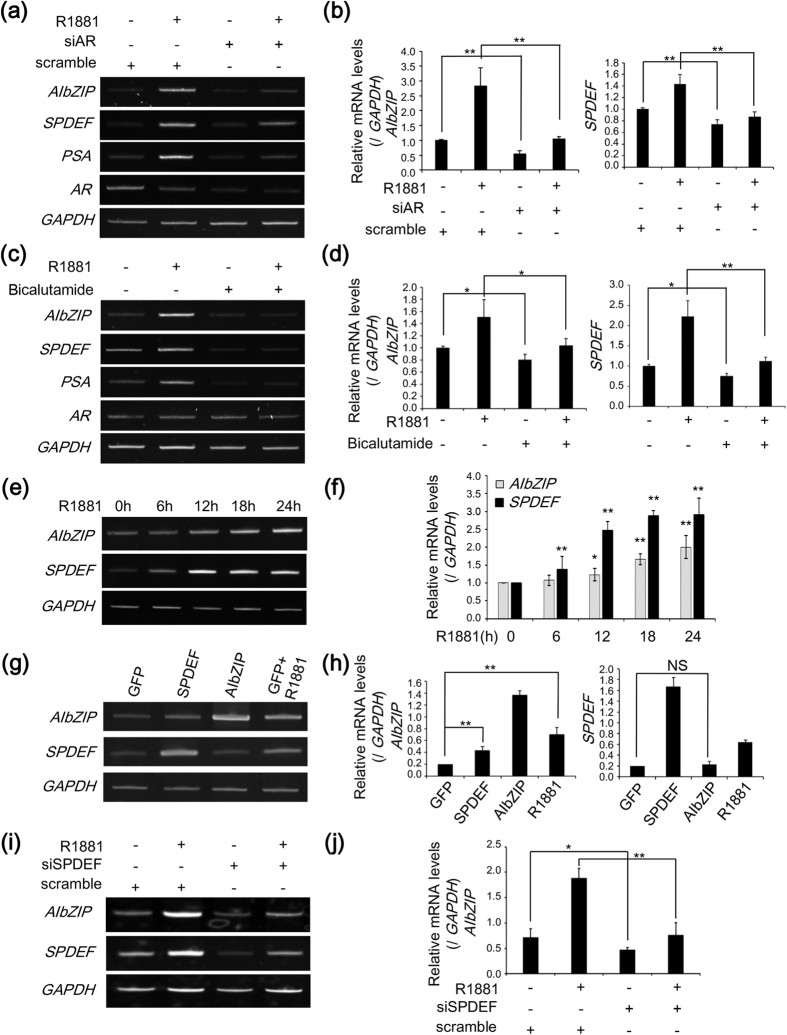
*AIbZIP* is induced by SPDEF acting downstream of AR. (**a**) RT-PCR analysis for *AIbZIP*, *SPDEF*, *PSA*, and *AR* in LNCaP cells transfected with siRNA targeting *AR* or scramble, and then treated with R1881 for 24 h. (**b**) Quantification for relative mRNA levels of *AIbZIP* and *SPDEF* in (**a**) (mean ± s.d., n = 3; **P < 0.01). (**c**) RT-PCR analysis for *AIbZIP*, *SPDEF*, *PSA*, and *AR* in LNCaP cells treated with R1881 and/or bicalutamide for 24 h. (**d**) Quantification for relative mRNA levels of *AIbZIP* and *SPDEF* in (**c**) (mean ± s.d., n = 3; *P < 0.05, **P < 0.01). (**e**) RT-PCR analysis for *AIbZIP* and *SPDEF* in LNCaP cells treated with R1881 for indicated time periods. (**f**) Quantification for relative mRNA levels of *AIbZIP* and *SPDEF* in (**e**) (mean ± s.d., n = 3; *P < 0.05, **P < 0.01, vs. without treatment). (**g**) RT-PCR analysis for *AIbZIP* and *SPDEF* in LNCaP cells transfected with indicated expression vectors by electroporation, or with a vector expressing GFP prior to treatment with R1881 for 24 h. A vector expressing GFP was used as a control vector. (**h**) Quantification for relative mRNA levels of *AIbZIP* and *SPDEF* in (**g**) (mean ± s.d., n = 3; **P < 0.01, NS; non-significant). (**i**) RT-PCR analysis for *AIbZIP* and *SPDEF* in LNCaP cells transfected with siRNA targeting *SPDEF* or scramble, and treated with R1881 for 24 h. (**j**) Quantification for relative mRNA levels of *AIbZIP* in (**i**) (mean ± s.d., n = 3; *P < 0.05, **P < 0.01). Full-length gels are presented in Supplementary Figure S9.

Next, we analyzed the mechanisms responsible for the upregulation of *AIbZIP* expression. The AR activated by androgen binds to androgen response elements (AREs) in the promoter regions of target genes^23^,^24^. Knockdown of *AR* suppressed the transcriptional induction of its target gene *PSA* by R1881 (Fig. 2a,b). Similarly, *AIbZIP* expression induced by R1881 was significantly reduced in *AR*-knockdown cells (Fig. 2a,b). In addition, the AR inhibitor bicalutamide also suppressed the transcriptional induction of *AIbZIP* and *PSA* in LNCaP cells (Fig. 2c,d), indicating that the AR is indispensable for the upregulation of *AIbZIP* by androgen stimulation. However, no exact sequence of AREs exists in the promoter region of *AIbZIP*, suggesting that the AR does not directly activate transcription of *AIbZIP*. To disclose the leading molecule for transcription of *AIbZIP*, we focused on SAM pointed domain-containing ETS transcription factor (SPDEF). It was shown that SPDEF promotes transcription of *PSA* through direct binding to the *PSA* promoter region, and interacts with AR to cooperatively enhance *PSA* promoter activity^25^. *SPDEF* has also been reported to be upregulated by R1881 stimuli in LNCaP cells^26^. Indeed, knockdown of *AR* or treatment with bicalutamide suppressed the transcriptional induction of *SPDEF* by R1881 treatment (Fig. 2a–d). Moreover, *SPDEF* mRNA levels were increased at an earlier time point compared with *AIbZIP* mRNA in LNCaP cells treated with R1881 (Fig. 2e,f). Therefore, we hypothesized that SPDEF mediates the androgen-induced transcriptional regulation of *AIbZIP*. Overexpression of AIbZIP had no effect on the expression levels of *SPDEF*, while LNCaP cells expressing SPDEF showed a 2.5-fold increase in *AIbZIP* expression (Fig. 2g,h). Alternatively, knockdown of *SPDEF* in LNCaP cells dramatically decreased the transcriptional induction of *AIbZIP* by R1881 treatment (Fig. 2i,j). These results supported our hypothesis that *AIbZIP* is upregulated by SPDEF, which acts downstream of AR.

### SPDEF directly binds to the promoter region of *AIbZIP*

**Figure 3:**
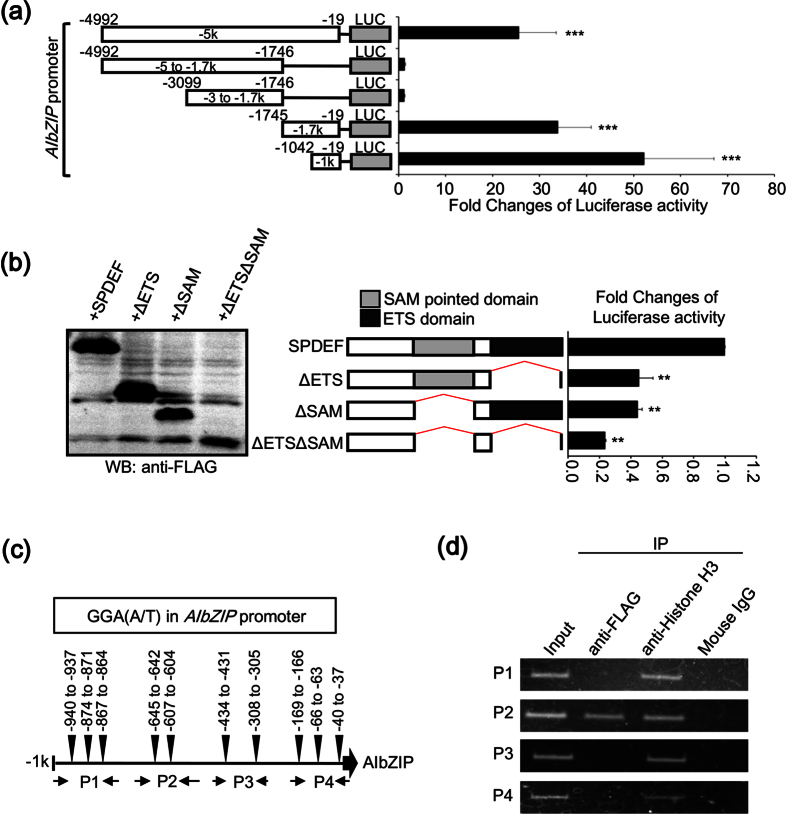
SPDEF directly binds to the promoter region of *AIbZIP.* (**a**) Left: schematic representation of reporter constructs containing indicated promoter regions upstream of the human *AIbZIP* gene. Luc: luciferase reporter gene. Right: luciferase reporter analysis using HEK293T cells co-transfected with each reporter construct and a vector expressing FLAG-tagged SPDEF (mean ± s.d., n = 6; ***P < 0.001, vs. pGL3-basic reporter plasmid without a promoter region). (**b**) Left: WB analysis for the expression of truncated SPDEF mutants using anti-FLAG antibody. SPDEF: FLAG-tagged full-length SPDEF, ΔETS: FLAG-tagged SPDEF lacking the ETS domain, ΔSAM: FLAG-tagged SPDEF lacking the SAM pointed domain, ΔETSΔSAM: FLAG-tagged SPDEF lacking both the ETS and the SAM pointed domains. Middle: schematic representation for each SPDEF mutant construct. The SAM pointed domain and the ETS domain are indicated. Right: luciferase reporter analysis using HEK293T cells co-transfected with a reporter construct containing −1 kb upstream of *AIbZIP* and vectors expressing a series of truncated SPDEF mutants (mean ± s.d., n = 3; **P < 0.01, vs. SPDEF). (**c**) Schematic representation of −1 kb upstream of *AIbZIP*. Ten GGA(A/T) core consensus sequences (−940 to −937 bp, −874 to −871 bp, −867 to −864 bp, −645 to −642 bp, −607 to −604 bp, −434 to −431 bp, −308 to −305 bp, −169 to −166 bp, −66 to −63 bp, −40 to −37 bp), and the annealing sites of each primer set (P1-P4) used in ChIP assays are indicated. (**d**) LNCaP cells were infected with a vector expressing FLAG-tagged SPDEF for 72 h before immunoprecipitation of the chromatin with anti-FLAG or anti-Histone H3 antibodies. Mouse IgG was used as a negative control. The purified input DNAs and the immunoprecipitated DNAs were analyzed by PCR using the primer sets shown in (**c**). Full-length gels and blots are presented in Supplementary Figure S10.

To examine whether SPDEF directly induces *AIbZIP*, we conducted reporter assays using a luciferase reporter gene driven by a 5-kb fragment of the promoter region of *AIbZIP*. Reporter activity was markedly enhanced (33-fold) by SPDEF expression (Fig. 3a). A serial deletion analysis showed that the region between −1042 and −19 confers the maximal promoter activity of *AIbZIP* (Fig. 3a). Moreover, deletion of the DNA-binding domains of SPDEF resulted in a decrease of the promoter activity induced by SPDEF (Fig. 3b). The ETS family proteins contain an evolutionarily conserved DNA-binding domain, which mediates binding to conserved purine-rich DNA sequences with a GGA(A/T) core consensus sequence^25^,^27^. We found that this core sequence is distributed in the promoter region of *AIbZIP* (Fig. 3c and Supplementary Figure S2). To narrow down the binding region of SPDEF, we designed four primer sets (P1–4) (Fig. 3c) and performed chromatin immunoprecipitation (ChIP) assays. Specific amplification was only detected by PCR analysis using primer set P2 (Fig. 3d). Collectively, these data indicated that SPDEF induced by androgen treatment regulates *AIbZIP* transcription through direct binding to the promoter region (−686 to −570) of endogenous *AIbZIP*, which contains two GGA(A/T) core sequences.

### AIbZIP is involved in prostate cancer cell proliferation

**Figure 4:**
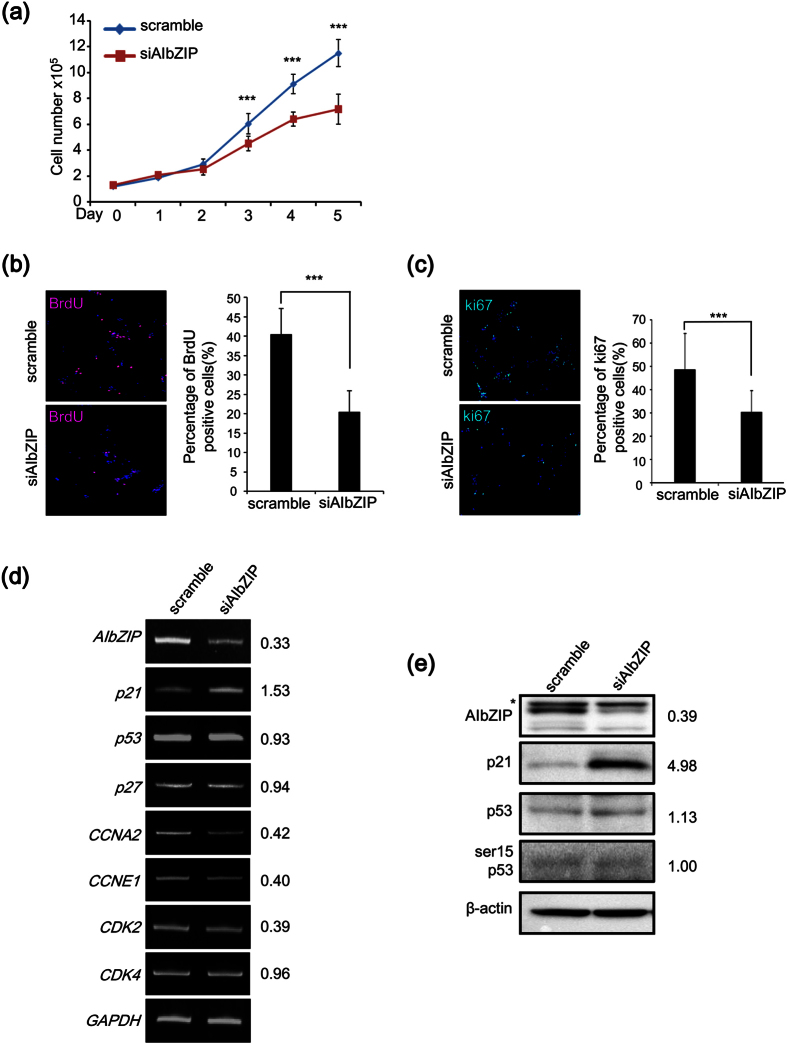
AIbZIP is involved in the proliferation of androgen-sensitive prostate cancer cells. (**a**) LNCaP cells were transfected with siRNA targeting *AIbZIP* or scramble, and the numbers of cells were counted for 5 days (means ± s.d., n = 4; ***P < 0.001, vs. scramble). (**b**) LNCaP cells transfected with siRNA targeting *AIbZIP* or scramble were immunostained with anti-BrdU antibody. BrdU: red, DAPI: blue, overlaps: magenta. Right graph shows the percentage of BrdU-positive cells (means ± s.d., scramble; n = 12, siAIbZIP; n = 16; ***P < 0.001). (**c**) LNCaP cells transfected with siRNA targeting *AIbZIP* or scramble were immunostained with anti-Ki67 antibody. Ki67: green, DAPI: blue, and overlaps: aqua. Right graph shows the percentage of Ki67-positive cells (means ± s.d., scramble; n = 11, siAIbZIP; n = 19; ***P < 0.001). (**d**) RT-PCR analysis for *AIbZIP* and cell cycle-related genes in LNCaP cells transfected with siRNA targeting *AIbZIP* or scramble. The numbers represent fold changes of mRNA levels (n = 3). *p21*: cyclin dependent kinase inhibitor 1, *p27*: cyclin dependent kinase inhibitor 1B, *CCNA2*: cyclin A2, *CCNE1*: cyclin E1, *CDK2*: cyclin-dependent kinase 2, *CDK4*: cyclin-dependent kinase 4, *p53*: tumor protein p53. (**e**) WB analysis for AIbZIP, p21, p53, and phosphorylated p53 (ser15 p53) in LNCaP cells transfected with siRNA targeting *AIbZIP* or scramble. The numbers represent fold changes of protein levels (n = 3). Asterisk: nonspecific bands. Full-length gels and blots are presented in Supplementary Figure S11.

Next, we investigated whether AIbZIP is involved in the proliferation of prostate cancer cells. We performed knockdown of *AIbZIP* in LNCaP cells using a mixture of three small interfering RNAs (siRNAs) (Supplementary Figure S3), and then counted the number of cells for 5 days. Without R1881 treatment, the number of cells was significantly reduced by 39.6% in *AIbZIP*-knockdown cells compared with control cells on day 5 (Fig. 4a). Incorporation of 5-Bromo-2′-deoxyuridine (BrdU), which determines the frequency of cells undergoing DNA synthesis and division, was dramatically decreased in *AIbZIP*-knockdown cells (Fig. 4b). Furthermore, the percentage of cells positive for the cancer proliferation marker Ki67 was also significantly decreased by *AIbZIP*-knockdown (Fig. 4c), indicating that knockdown of *AIbZIP* suppresses the proliferation of LNCaP cells.

To address the mechanism responsible for the antiproliferative effects of *AIbZIP* silencing, we examined the effects of *AIbZIP*-knockdown on the expression of cell cycle-related genes. Following *AIbZIP* knockdown, the mRNA levels of *cyclin A2 (CCNA2*), *cyclin E1 (CCNE1*) and *cyclin-dependent kinase 2 (CDK2*) were reduced compared with control sample (Fig. 4d). In contrast, both the mRNA and protein expression levels of *cyclin dependent kinase inhibitor 1 (p21*) were highly upregulated in *AIbZIP*-knockdown cells, although the phosphorylation of tumor protein p53 (p53) was unaffected (Fig. 4d,e), suggesting that silencing of *AIbZIP* upregulates the expression of *p21* independently of p53. p21 is a major molecule to inhibit the activities of cyclin-CDK complexes and negatively modulates cell cycle progression^28^. We therefore focused on p21 especially among the cell cycle-related genes whose expressions were affected by *AIbZIP*-knockdown.

**Figure 5:**
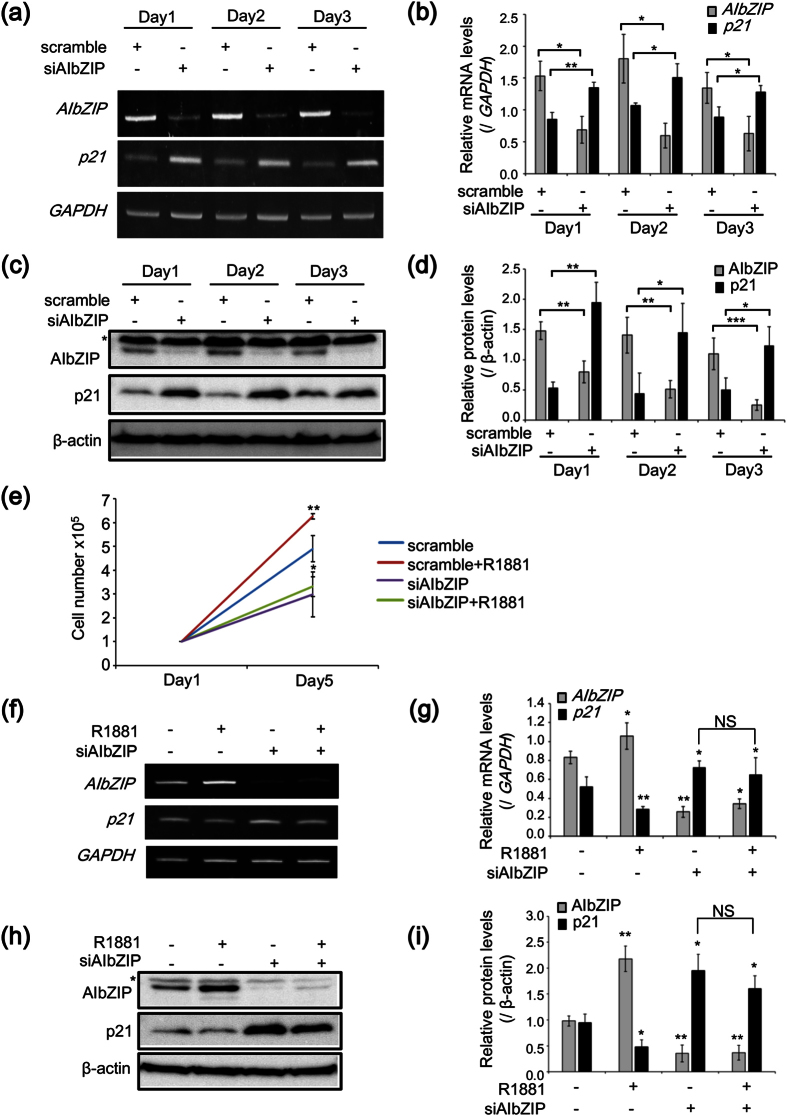
AIbZIP suppresses *p21* expression to promote the proliferation of prostate cancer cells induced by AR signaling. (**a**) RT-PCR analysis for *AIbZIP* and *p21* in LNCaP cells at indicated days after transfection with siRNA targeting *AIbZIP* or scramble. (**b**) Quantification for relative mRNA levels of *AIbZIP* and *p21* in (**a**) (mean ± s.d., n = 3; *P < 0.05, **P < 0.01). (**c**) WB analysis for AIbZIP and p21 in LNCaP cells at indicated days after transfection with siRNA targeting *AIbZIP* or scramble. Asterisk: nonspecific bands. (**d**) Quantification for relative protein levels of AIbZIP and p21 in (**c**) (mean ± s.d., n = 3; *P < 0.05, **P < 0.01, ***P < 0.001). (**e**) LNCaP cells were transfected with siRNA targeting *AIbZIP* or scramble, and then treated with 0.1 nM R1881 for 5 days. Cell numbers were counted on days 1 and 5 (mean ± s.d., n = 3; *P < 0.05, **P < 0.01, vs. scramble). (**f**) RT-PCR analysis for *AIbZIP* and *p21* in LNCaP cells transfected with siRNA targeting *AIbZIP* or scramble, and then treated with 0.1 nM R1881 for 5 days. (**g**) Quantification for relative mRNA levels of *AIbZIP* and *p21* in (**f**) (mean ± s.d., n = 3; *P < 0.05, **P < 0.01, vs. scramble and without treatment, NS; non-significant). (**h**) WB analysis for AIbZIP and p21 in LNCaP cells transfected with siRNA targeting *AIbZIP* or scramble, and then treated with 0.1 nM R1881 for 5 days. Asterisk: nonspecific bands. (**i**) Quantification for relative protein levels of AIbZIP and p21 in (**h**) (mean ± s.d., n = 3; *P < 0.05, **P < 0.01, vs. scramble and without treatment, NS; non-significant). Full-length gels are presented in Supplementary Figure S12.

To confirm that AIbZIP regulates the expression of *p21*, we examined the changes in *p21* expression patterns after transfection with siRNA targeting *AIbZIP*. The expression of *AIbZIP* was effectively suppressed for 3 days (Fig. 5a–d), while the expression pattern of *p21* was inversely correlated with that of *AIbZIP* (Fig. 5a–d). We also performed an additional proliferation assay using *AIbZIP*-knockdown or control cells with or without R1881 treatment. R1881 stimuli induced a reliable increase in the proliferation of control cells (Fig. 5e), but failed to rescue the suppression of cell proliferation by *AIbZIP*-knockdown (Fig. 5e). Consistently, the increase of *p21* expression in *AIbZIP*-knockdown cells was not affected by R1881 treatment, although R1881 stimuli decreased *p21* expression in control cells (Fig. 5f–i). In contrast, overexpression of AIbZIP dramatically increased the proliferation of LNCaP cells without R1881 treatment (Supplementary Figure S4). These results indicated that AIbZIP may regulate LNCaP cell proliferation induced by AR signaling via inhibition of *p21* expression.

### AIbZIP represses *p21* expression via prevention of OASIS activation

**Figure 6:**
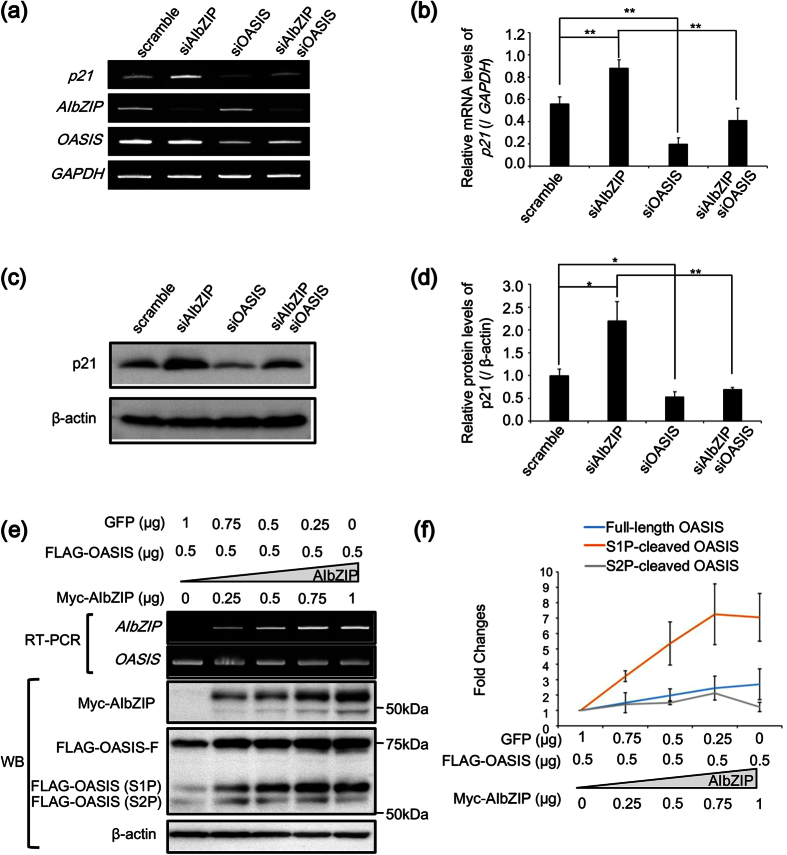
AIbZIP represses *p21* expression via prevention of OASIS activation. (**a**) RT-PCR analysis for *p21*, *AIbZIP* and *OASIS* in LNCaP cells transfected with siRNA targeting *AIbZIP*, *OASIS*, or scramble. (**b**) Quantification for relative mRNA levels of *p21* in (**a**) (mean ± s.d., n = 3; **P < 0.01). (**c**) WB analysis for p21 in LNCaP cells transfected with siRNA targeting *AIbZIP*, *OASIS*, or scramble. (**d**) Quantification for relative protein levels of p21 in (**c**) (mean ± s.d., n = 3; *P < 0.05, **P < 0.01). (**e**) RT-PCR and WB analysis for *AIbZIP* and *OASIS* in HEK293T cells co-transfected with indicated amounts of vectors expressing FLAG-tagged OASIS (FLAG-OASIS), Myc-tagged AIbZIP (Myc-AIbZIP), and GFP. A vector expressing GFP was used to adjust the total amounts of vectors to 1.5 μg. FLAG-OASIS-F: FLAG-tagged full length OASIS, FLAG-OASIS (S1P): FLAG-tagged S1P-cleaved OASIS, FLAG-OASIS (S2P): FLAG-tagged S2P-cleaved OASIS. (**f**) Fold changes in expression levels of full-length, S1P-, and S2P-cleaved OASIS in (**e**). Full-length gels and blots are presented in Supplementary Figure S13.

The next issue is how AIbZIP promotes cell proliferation through regulation of *p21* expression. Interestingly, the bZIP transcription factor OASIS, which is structurally similar to AIbZIP (Fig. 1e), has been reported to directly activate *p21* transcription^29^. We investigated the expression levels of *OASIS* in several cell lines. Unexpectedly, *OASIS* was strongly expressed in PC-3 and Caco-2 cells, in which *AIbZIP* was hardly expressed (Fig. 1b and Supplementary Figure S5). These data suggested that the pattern of *OASIS* expression in various cancer cell lines is not correlated with that of *AIbZIP*. However, *OASIS* demonstrated moderate expression in LNCaP cells (Supplementary Figure S5), and knockdown of *OASIS* significantly decreased p21 expression in LNCaP cells (Fig. 6a–d). Moreover, knockdown of *AIbZIP* alone increased p21 mRNA and protein, and this increase was blocked by simultaneous knockdown of *OASIS* (Fig. 6a–d), suggesting that AIbZIP modulates the function of OASIS to regulate *p21* expression, at least in LNCaP cells. The reason why *AIbZIP* knockdown induced p21 protein more than its mRNA remains unknown, but it is well known that the stability of p21 protein is increased when cell cycle progression is negatively regulated^30^,^31^. Therefore, it is conceivable that the apparent induction of p21 protein was much higher than that of its mRNA. To identify how AIbZIP affects the function of OASIS, we expressed a constant amount of FLAG-OASIS and various amounts of Myc-tagged full-length AIbZIP (Myc-AIbZIP) in HEK293T cells and analyzed the effects on the activation of OASIS. RT-PCR showed the mRNA levels of *OASIS* were not affected by any amounts of AIbZIP (Fig. 6e). OASIS is activated by sequential cleavage by S1P and S2P at the Golgi apparatus and then completely cleaved N-terminal fragments translocate to the nucleus to promote gene transcription^15^ (Supplementary Figure S6). Interestingly, WB analysis showed that the amounts of S1P-cleaved OASIS were strongly increased by AIbZIP in a dose-dependent manner (Fig. 6e,f). However, the amounts of full-length and S2P-cleaved OASIS remained relatively unchanged (Fig. 6e,f), suggesting that AIbZIP does not affect S1P-mediated cleavage of OASIS at the Golgi apparatus, but could prevent S2P-mediated cleavage, thereby preventing release of the N-terminus of OASIS from the Golgi apparatus, resulting in inhibition of *p21* transcription by OASIS.

### AIbZIP prevents S2P-mediated cleavage of OASIS via direct interaction with OASIS at the Golgi apparatus

**Figure 7:**
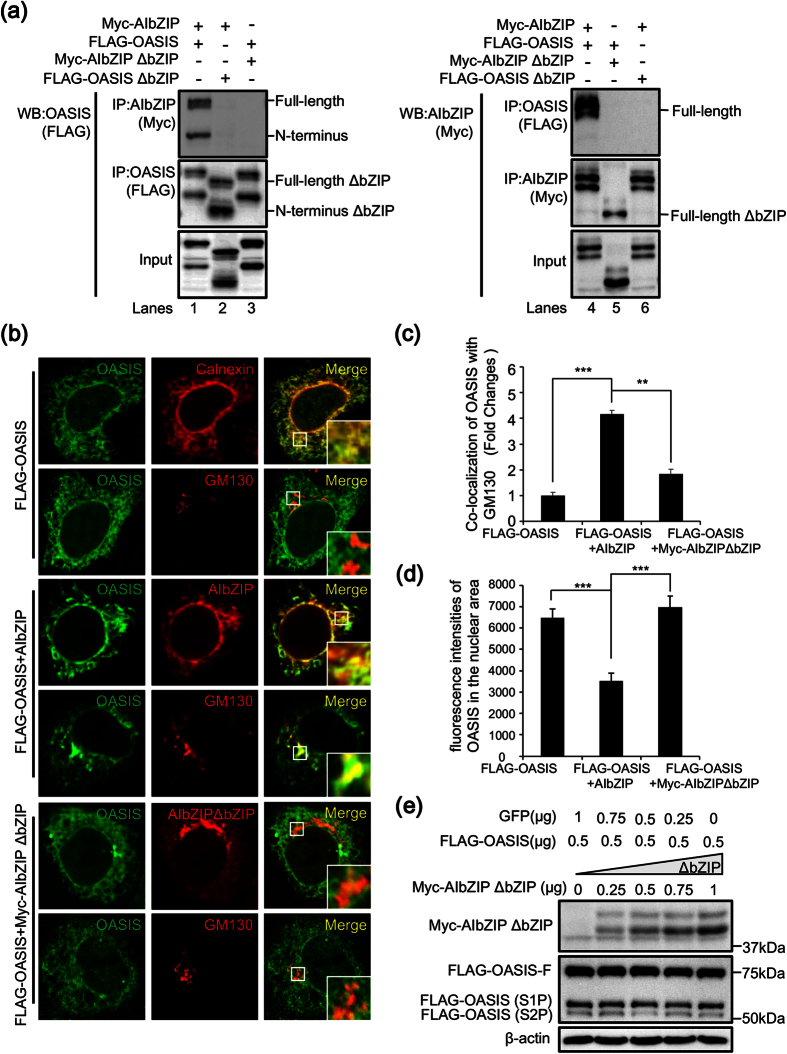
AIbZIP prevents S2P-mediated cleavage of OASIS via direct interaction with OASIS at the Golgi apparatus. (**a**) Immunoprecipitation using HEK293T cells transfected with indicated expression vectors. Left: cell lysates were immunoprecipitated using anti-Myc or anti-FLAG antibodies, and the precipitates were subjected to WB analysis using anti-FLAG antibody. Right: cell lysates were immunoprecipitated using anti-FLAG or anti-Myc antibodies, and the precipitates were subjected to WB analysis using anti-Myc antibody. IP: immunoprecipitation, ΔbZIP: lacking the bZIP domain. (**b**) Top two panels: HeLa cells were transfected with vectors expressing FLAG-OASIS for 24 h, then co-stained with anti-FLAG (OASIS) and anti-calnexin or anti-GM130 antibodies. Middle two panels: HeLa cells were co-transfected with vectors expressing FLAG-OASIS and AIbZIP for 24 h, then co-stained with anti-FLAG (OASIS) and anti-AIbZIP or anti-GM130 antibodies. Bottom two panels: HeLa cells were co-transfected with vectors expressing FLAG-OASIS and Myc-AIbZIP ΔbZIP for 24 h, then co-stained with anti-FLAG (OASIS) and anti-AIbZIP or anti-GM130 antibodies. Insert large square is high-magnification view of the small frame in each image. Note that the signals of OASIS were detected from the ER, and partly from the Golgi apparatus and nucleus in cells transfected with FLAG-OASIS alone. Co-transfection with FLAG-OASIS and AIbZIP increased the signals of OASIS in the Golgi apparatus, and decreased them in the nucleus. Alternatively, co-transfection with FLAG-OASIS and Myc-AIbZIP ΔbZIP had no effect on the subcellular localization of OASIS. (**c**) Quantification of co-localization between OASIS and GM130 in (**b**). The details for quantification was described in Methods section (mean ± s.d., FLAG-OASIS; n = 19, FLAG-OASIS + AIbZIP; n = 14, FLAG-OASIS + Myc-AIbZIP ΔbZIP; n = 18; **P < 0.01, ***P < 0.001). (**d**) Quantification of fluorescence intensities in the nuclear area in (**b**). The y-axis represents the average of measured values (mean ± s.d., FLAG-OASIS; n = 18, FLAG-OASIS + AIbZIP; n = 16, FLAG-OASIS + Myc-AIbZIP ΔbZIP; n = 20; ***P < 0.001). (**e**) WB analysis for AIbZIP and OASIS in HEK293T cells co-transfected with indicated amounts of vectors expressing FLAG-OASIS, Myc-AIbZIP ΔbZIP, and GFP. FLAG-OASIS-F: FLAG-tagged full length OASIS, FLAG-OASIS (S1P): FLAG-tagged S1P-cleaved OASIS, FLAG-OASIS (S2P): FLAG-tagged S2P-cleaved OASIS. Full-length blots are presented in Supplementary Figure S14.

bZIP family proteins are known to form homo- or heterodimers to switch their target genes^16^,^32^. Hence, we performed co-immunoprecipitation assays using HEK293T cells co-transfected with Myc-AIbZIP and FLAG-OASIS to confirm whether these two molecules interact with each other. Full-length and N-terminal OASIS co-precipitated with full-length AIbZIP (Fig. 7a, lane 1), and alternatively full-length AIbZIP co-precipitated with full-length and N-terminal OASIS (Fig. 7a, lane 4). To determine the interaction sites of both AIbZIP and OASIS, we constructed mutant vectors expressing full-length AIbZIP or OASIS lacking the bZIP domain (Myc-AIbZIP ΔbZIP or FLAG-OASIS ΔbZIP). Co-immunoprecipitation showed that FLAG-OASIS was not co-precipitated with Myc-AIbZIP ΔbZIP (Fig. 7a, lanes 3 and 5), and Myc-AIbZIP was not co-precipitated with FLAG-OASIS ΔbZIP (Fig. 7a, lanes 2 and 6), indicating that full-length AIbZIP interacts with full-length and N-terminal OASIS through their respective bZIP domains.

To assess the impact of the interaction of AIbZIP with full-length and N-terminal OASIS on their functions, we analyzed their subcellular localizations. When FLAG-OASIS was introduced alone, it was localized mainly in the ER, but also partly in the Golgi apparatus and nucleus (Fig. 7b,c). Interestingly, when AIbZIP and FLAG-OASIS were co-transfected, both OASIS and AIbZIP accumulated at a juxtanuclear, Golgi apparatus-like region (Fig. 7b). Immunostaining for OASIS and GM130 showed the majority of OASIS signals overlapped with GM130 (Fig. 7b,c). Furthermore, FLAG-OASIS signals in the nucleus were reduced by co-transfection with AIbZIP (Fig. 7b,d). When FLAG-OASIS was co-transfected with Myc-AIbZIP ΔbZIP, the signals of Myc-AIbZIP ΔbZIP were also detected from ER- and Golgi apparatus-like regions (Fig. 7b). However, the signals of OASIS hardly overlapped with Myc-AIbZIP ΔbZIP and GM130 (Fig. 7b,c), and the nuclear signals of OASIS were unchanged (Fig. 7b,d). This observation indicated AIbZIP could cause the accumulation of both full-length and N-terminal OASIS at the Golgi apparatus by direct binding of each other, resulting in the inhibition of N-terminal OASIS translocation to the nucleus. To examine whether the interaction of AIbZIP and OASIS affects cleavage of OASIS, we co-transfected HEK293T cells with FLAG-OASIS and Myc-AIbZIP ΔbZIP. Interestingly, in contrast to the results in Fig. 6e,f, the amounts of full-length, S1P-, and S2P-cleaved N-terminal OASIS were not affected by any amounts of Myc-AIbZIP ΔbZIP, which could not interact with OASIS (Fig. 7e and Supplementary Figure S7). These results suggested that AIbZIP prevents OASIS activation by inhibiting S2P-cleavage through direct binding at the Golgi apparatus.

## Discussion

**Figure 8:**
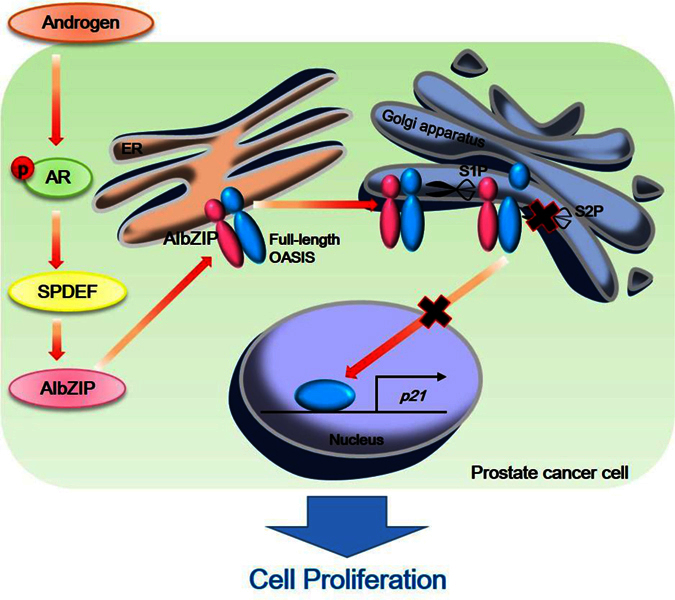
Schematic representation of the summary. Androgen receptor activated by androgen induces SPDEF expression. Then SPDEF promotes transcription of *AIbZIP* through direct binding to the GGA(A/T) sequence within the −1 kb upstream of *AIbZIP*. Full-length AIbZIP interacts with both full-length and S1P-cleaved OASIS and inhibits S2P-mediated cleavage of OASIS, which reduces the translocation of OASIS to the nucleus to promote transcription of *p21*.

AR signaling plays critical roles in the development, proliferation, survival, and progression of prostate cancer^4^. Understanding the physiological functions of downstream molecules of AR is crucial for the development of effective therapeutic strategies targeting prostate cancer. In this study, we demonstrated that AIbZIP is a key molecule of the AR axis regulating the proliferation of prostate cancer by the following evidence: (1) AIbZIP was highly expressed in androgen-sensitive LNCaP cells but not in androgen-insensitive PC-3 cells; (2) *AIbZIP* was upregulated by SPDEF, which acts downstream of AR signaling; (3) knockdown of *AIbZIP* decreased the proliferation of LNCaP cells while overexpression of AIbZIP increased it; (4) AIbZIP prevented OASIS from promoting transcription of its target gene *p21*; (5) AIbZIP directly bound with OASIS via the bZIP domain, and inhibited S2P-mediated cleavage of OASIS (Fig. 8). To date, treatments for the prostate cancer are focused on the reduction of serum androgen levels and inhibition of AR^33^,^34^. However, androgen deprivation therapy (ADT) increases the sensitivity of AR to androgen, meaning that the AR signal becomes hyperactivated in response to the trace amounts of androgen^33^,^34^. Therefore, our findings provide a great possibility that targeting AIbZIP could be a more effective therapy for androgen-sensitive prostate cancer than ADT because AIbZIP is an essential factor for the proliferation of these cancer cells. Notably, we demonstrated that overexpression of AIbZIP significantly increased the proliferation of LNCaP cells without androgen stimulation, implying that the increase of AIbZIP expression could contribute to androgen-independent growth of prostate cancer cells and/or acquired resistance to ADT. Indeed, AIbZIP expression has been reported in all grades of prostate cancer^35^, and the expression levels of AIbZIP are increased in malignant tissue compared with benign tissue^17^,^18^. It is conceivable that interfering with AIbZIP expression or its function could effectively control the proliferation of prostate cancer cells and reduce the side effects of ADT.

Our study demonstrated that the AR axis upregulates the expression of *SPDEF*, which in turn SPDEF directly promotes transcription of *AIbZIP*. It is well known that SPDEF is highly expressed in androgen-sensitive prostate cancer cells LNCaP and LNCaP C4-2, but depleted in androgen-insensitive cells PC-3 and DU-145^36^,^37^,^38^. These expression patterns are consistent with those of AIbZIP. Furthermore, it was reported that knockdown of *SPDEF* decreased the proliferation rates of LNCaP and LNCaP C4-2 cells^38^,^39^, the data of which support our present results. The expression of genes relevant for cell proliferation including insulin-like growth factors (*IGF*-*I*, *IGF*-*II*) and the IGF-receptor (*IGFR*) are considered to be regulated downstream of SPDEF^38^,^39^, but the significance of these genes in the proliferation of prostate cancer cells remain unclear. Knockdown of *AIbZIP*, which is a direct target of SPDEF, suppressed the proliferation of prostate cancer cells, indicating that AIbZIP functions downstream of SPDEF as a major regulator of proliferation in these cells. However, several groups reported that SPDEF is a negative regulator of cell proliferation in some cancer types including prostate cancer^40^,^41^. It was shown that overexpression of SPDEF in PC-3 cells inhibited proliferation and induced apoptosis via negative regulation of stathmin and survivin expression^36^,^41^. Taken together with our findings, SPDEF has different roles in the regulation of androgen-sensitive and -insensitive prostate cancer cell proliferation. Further investigation of the roles of SPDEF in several cancers including androgen-insensitive prostate cancer is required to understand the mechanism leading to its contradictory effects on cell proliferation.

AIbZIP is structurally a transcription factor that belongs to the OASIS family^11^. However, it displays some features that differ from other OASIS family members. First, AIbZIP was localized to the Golgi apparatus as well as the ER under normal conditions. Second, AIbZIP was never cleaved in response to various stimuli including ER stress although it has the potential to be cleaved by S1P and S2P. These findings indicated that AIbZIP may function as the full-length form at the Golgi apparatus. A previous study of the interaction between bZIP transcription factors using protein arrays showed that AIbZIP could interact with other OASIS family members^42^. However, the physiological significance of those complexes has not been elucidated. Our data showed that AIbZIP may prevent S2P-mediated cleavage of OASIS to inhibit its nuclear translocation via interaction with OASIS. It provides a new insight that AIbZIP could negatively modulate the activation of other bZIP transmembrane proteins by forming a heterodimer at the Golgi apparatus. However, the mechanism by which AIbZIP inhibits protease processing remains unclear. Given that AIbZIP can bind to OASIS via their bZIP domains, one possibility is that this binding may cause a conformational change in the transmembrane domain of OASIS that conceals the S2P recognition site. Further studies are needed to clarify the detailed mechanism involved in the prevention of S2P-mediated cleavage by AIbZIP.

In conclusion, we demonstrated that *AIbZIP* is upregulated by SPDEF acting downstream of AR in prostate cancer cells. AIbZIP tightly suppresses *p21* expression by inhibiting OASIS activation to promote LNCaP cell proliferation. These findings provide a new possibility that AIbZIP could be a potential cancer therapeutic target of prostate cancer.

## Methods

### Cell culture and reagents

LNCaP cells were grown in Roswell Park Memorial Institute medium-1640 (RPMI1640; Gibco) containing 10% fetal calf serum (FCS). HeLa, HEK293T, MCF7, and PC-3 cells were grown in Dulbecco’s modified Eagle’s medium (DMEM; Gibco) containing 10% FCS. U251MG cells were grown in Eagle’s minimal essential medium (EMEM; DS Pharma) containing 10% FCS. Caco-2 cells were grown in EMEM containing 20% FCS. Plat-A cells were grown in DMEM containing 10% FCS supplemented with 1 μg/mL puromycin (Sigma-Aldrich) and 10 μg/mL blasticidin (Invitrogen). R1881 and bicalutamide were purchased from Wako. Thapsigargin, tunicamycin and brefeldin A were purchased from Sigma-Aldrich. Unless otherwise stated, thapsigargin, tunicamycin, brefeldin A, R1881, and bicalutamide were used at the concentration of 1 μM, 3 μg/ml, 1 μM, 1 nM, and 10 μM respectively.

### Plasmids, transfection and electroporation

*AIbZIP* and *SPDEF* cDNAs were cloned from LNCaP mRNA by using PCR, and inserted into the pMX retroviral vector, the pTRE2hyg tet-off expression vector, or the pcDNA3.1(+) expression vector. The expression vectors for FLAG/Myc-AIbZIP, and Myc-AIbZIP ΔbZIP were amplified by PCR using full-length AIbZIP as a template. The expression vectors for FLAG-SPDEF and truncated mutants of FLAG-SPDEF were constructed from full-length SPDEF by PCR. The expression vector for FLAG-OASIS was described in previous our report^43^. The expression vector for FLAG-OASIS ΔbZIP was constructed using FLAG-OASIS plasmid. The transfections were performed using ScreenFect A (Wako) according to the manufacturer’s protocol. The transfections of LNCaP cells in ChIP assays were performed using electroporation (BEX CUY21EX). The reporter plasmids, driven by the human *AIbZIP* promoter regions, were cloned from LNCaP genome by using PCR and inserted into pGL3-basic reporter plasmids. All primer sets for cloning are indicated in Supplementary Table 1.

### Virus Infection

The pMX retroviral vectors harboring the *AIbZIP* and *SPDEF* were introduced into Plat-A retrovirus packaging cells. LNCaP cells were infected with viral supernatant mixed with polybrane (4 μg/ml) for 24 h.

### Tet-off system

LNCaP cells were transfected with pTet-Off Advanced vector (Clontech). A stable transformant was isolated by 800 μg/ml G418 (Wako), and tested for induction according to the manufacturer’s protocol. LNCaP tet-off cells were maintained in RPMI1640 medium with 10% FCS containing 400 μg/ml G418 and 1 μg/ml doxycycline (Clontech). LNCaP tet-off cells were transfected with pTRE2hyg-FLAG-AIbZIP, selected using 1 mg/ml hygromycin B (Wako), and maintained in RPMI 1640 medium with 10% FCS containing 400 μg/ml G418, 500 μg/ml hygromycin B and 1 μg/ml doxycycline.

### Western blot analysis and Antibodies

Western blot analysis was performed as described previously^44^. The quantification of western blot analysis was performed by Quantity one software (Bio-Rad). Antibodies used in western blot analysis, immunoprecipitation, and immunofluorescence were summarized in Supplementary Table 2.

### RNA extraction and RT-PCR

Total RNA was extracted from cells using ISOGEN (Nippongene) according to the manufacturer’s protocol. RT-PCR assays were performed according to our published procedures^44^. Primer sequences are summarized in Supplementary Table 3. The quantification of RT-PCR was performed by Quantity one software (Bio-Rad).

### Immunofluorescence staining

Immunofluorescence staining was performed as described previously^43^. Staining was visualized under a confocal microscope (Olympus FV1000D). The quantification of co-localization between OASIS and GM130 signals was calculated on a pixel-by-pixel basis WCIF version of ImageJ (http://www.uhnresearch.ca/facilities/wcif/imagej/), which generates a scatter-plot of the pixel intensities to calculate the threshold for each channel^45^. The scatter-plot is then used to calculate the number of co-localized pixels and their intensities. To calculate the proportion of co-localization, we used the sum of intensities greater than the threshold that showed co-localization divided by the sum of intensities greater than the threshold of the respective channel that did not co-localize. Fluorescence intensity in nuclear area was measured by Image_analysis_software CS Analyzer 4 (ATTO CORPORATION).

### RNA interference and Cell proliferation assay

LNCaP cells were transfected using Lipofectamine RNAiMAX (Invitrogen) according to the manufacturer’s protocols. The siRNA sequences were summarized in Supplementary Table 4. For cell proliferation assay, after treatment with doxycycline or transfection with siRNAs into LNCaP cells (1 × 10^5^ cells/well in 6-well plates), the numbers of cells were counted at the indicated time.

### Luciferase assay

Reporter plasmids containing the AIbZIP promoter region were co-transfected into HEK293T cells along with Renilla luciferase (internal control) and vectors expressing a series of truncated SPDEF mutant using Screenfect A (Wako). 24 hours after transfection, cells were harvested into passive lysis buffer (Promega), and dual luciferase activity was assayed with a GloMax Multi + Detection System (Promega). Reporter luciferase activity was normalized to the internal Renilla control activity.

### Chromatin Immunoprecipitation assay and Immunoprecipitation

The chromatin immunoprecipitation assay was performed as previously described^15^. Briefly, LNCaP cells were infected with FLAG-SPDEF and cross-linked using formaldehyde for 10 min at 37 °C. Then cells were lysed using SDS lysis buffer and sonicated (25 × 5-s sonication pulses at 10-s intervals). Equal amounts of chromatin from each sample were incubated overnight at 4 °C with anti-FLAG M2 or anti-Histone H3 antibody. Cross-linking was reversed (>6 h at 65 °C) and the DNA was purified by phenol–chloroform extraction and ethanol precipitation. The purified DNAs were subjected to PCR analysis using primer sets summarized in Supplementary Table 5. For immunoprecipitation, cell lysates were incubated with anti-FLAG or anti-Myc antibodies overnight, and then rotated with protein G-agarose (Invitrogen) at 4 °C for 3 hours. The agarose beads were washed with TNE buffer (10 mM Tris, 1 mM EDTA, and 150 mM NaCl).

### ONCOMINE microarray datasets for *AIbZIP* expression

The datasets included 353, 83, 7, 199, 292, 136, 132, 63, 43 and 13 malignant tissue samples of breast cancer, prostate cancer, brain cancer, ovarian cancer, colorectal cancer, uterine cancer, lung cancer, endometrial cancer, liver cancer, and stomach cancer.

### Statistical Analysis

Statistical comparisons were made using the unpaired Student’s t-test. Statistical significance between two samples was determined by a p-value of less than 0.05. p-values of less than 0.05, 0.01 or 0.001 are described as *p < 0.05; **p < 0.01; or ***p < 0.001, respectively.

## Additional Information

**How to cite this article**: Cui, X. *et al.* The androgen-induced protein AIbZIP facilitates proliferation of prostate cancer cells through downregulation of p21 expression. *Sci. Rep.* **6**, 37310; doi: 10.1038/srep37310 (2016).

**Publisher’s note**: Springer Nature remains neutral with regard to jurisdictional claims in published maps and institutional affiliations.

## Supplementary Material

Supplementary Information

## References

[b1] Lamb D. J., Weigel N. L. & Marcelli M. Androgen receptors and their biology. Vitam. Horm. 62, 199–230 (2001). 11345899 10.1016/s0083-6729(01)62005-3

[b2] Feldman B. J. & Feldman D. The development of androgen-independent prostate cancer. Nat. Rev. Cancer 1, 34–45, doi: 10.1038/35094009 (2001). 11900250

[b3] Tan M. H., Li J., Xu H. E., Melcher K. & Yong E. L. Androgen receptor: structure, role in prostate cancer and drug discovery. Acta. Pharmacol Sin. 36, 3–23, doi: 10.1038/aps.2014.18 (2015). 24909511 PMC4571323

[b4] Holzbeierlein J. *et al.* Gene expression analysis of human prostate carcinoma during hormonal therapy identifies androgen-responsive genes and mechanisms of therapy resistance. Am. J. Pathol. 164, 217–227, doi: 10.1016/S0002-9440(10)63112-4 (2004). 14695335 PMC1602218

[b5] Ron D. Translational control in the endoplasmic reticulum stress response. J. Clin. Invest. 110, 1383–1388, doi: 10.1172/JCI16784 (2002). 12438433 PMC151821

[b6] Schröder M. The unfolded protein response. Mol. Biotechnol. 34, 279–290, doi: 10.1385/MB:34:2:279 (2006). 17172673

[b7] Lu R., Yang P., O’Hare P. & Misra V. Luman, a new member of the CREB/ATF family, binds to herpes simplex virus VP16-associated host cellular factor. Mol. Cell Biol. 17, 5117–5126 (1997). 9271389 10.1128/mcb.17.9.5117 PMC232362

[b8] Nikaido T. *et al.* Expression of the novel transcription factor OASIS, which belongs to the CREB/ATF family, in mouse embryo with special reference to bone development. Histochem. Cell Biol. 116, 141–148, doi: 10.1007/s004180100279 (2001). 11685542

[b9] Kondo S. *et al.* BBF2H7, a novel transmembrane bZIP transcription factor, is a new type of endoplasmic reticulum stress transducer. Mol. Cell Biol. 27, 1716–1729, doi: 10.1128/MCB.01552-06 (2007). 17178827 PMC1820470

[b10] Omori Y. *et al.* CREB-H: a novel mammalian transcription factor belonging to the CREB/ATF family and functioning via the box-B element with a liver-specific expression. Nucleic Acids Res. 29, 2154–2162 (2001). 11353085 10.1093/nar/29.10.2154 PMC55463

[b11] Qi H. *et al.* AIbZIP, a novel bZIP gene located on chromosome 1q21.3 that is highly expressed in prostate tumors and of which the expression is up-regulated by androgens in LNCaP human prostate cancer cells. Cancer Res. 62, 721–733 (2002). 11830526

[b12] Yoshida H. *et al.* ATF6 activated by proteolysis binds in the presence of NF-Y (CBF) directly to the cis-acting element responsible for the mammalian unfolded protein response. Mol. Cell Biol. 20, 6755–6767 (2000). 10958673 10.1128/mcb.20.18.6755-6767.2000 PMC86199

[b13] Brown M. S., Ye J., Rawson R. B. & Goldstein J. L. Regulated intramembrane proteolysis: a control mechanism conserved from bacteria to humans. Cell 100, 391–398 (2000). 10693756 10.1016/s0092-8674(00)80675-3

[b14] Bailey D. & O’Hare P. Transmembrane bZIP transcription factors in ER stress signaling and the unfolded protein response. Antioxid Redox Signal 9, 2305–2321, doi: 10.1089/ars.2007.1796 (2007). 17887918

[b15] Kondo S. *et al.* OASIS, a CREB/ATF-family member, modulates UPR signalling in astrocytes. Nat. Cell Biol. 7, 186–194, doi: 10.1038/ncb1213 (2005). 15665855

[b16] Asada R., Kanemoto S., Kondo S., Saito A. & Imaizumi K. The signalling from endoplasmic reticulum-resident bZIP transcription factors involved in diverse cellular physiology. J. Biochem. 149, 507–518, doi: 10.1093/jb/mvr041 (2011). 21454302

[b17] Levesque M. H., El-Alfy M., Berger L., Labrie F. & Labrie C. Evaluation of AIbZIP and Cdc47 as markers for human prostatic diseases. Urology 69, 196–201, doi: 10.1016/j.urology.2006.11.001 (2007). 17270658

[b18] Schmidt U. *et al.* Quantitative multi-gene expression profiling of primary prostate cancer. Prostate 66, 1521–1534, doi: 10.1002/pros.20490 (2006). 16921506

[b19] Fujii T. *et al.* Use of stepwise subtraction to comprehensively isolate mouse genes whose transcription is up-regulated during spermiogenesis. EMBO Rep. 3, 367–372, doi: 10.1093/embo-reports/kvf073 (2002). 11943763 PMC1084061

[b20] Stelzer G. & Don J. Atce1: a novel mouse cyclic adenosine 3′,5′-monophosphate-responsive element-binding protein-like gene exclusively expressed in postmeiotic spermatids. Endocrinology 143, 1578–1588, doi: 10.1210/endo.143.5.8822 (2002). 11956138

[b21] Labrie C. *et al.* Androgen-regulated transcription factor AIbZIP in prostate cancer. J Steroid Biochem Mol Biol 108, 237–244, doi: 10.1016/j.jsbmb.2007.09.008 (2008). 17933519

[b22] Klausner R. D., Donaldson J. G. & Lippincott-Schwartz J. Brefeldin A: insights into the control of membrane traffic and organelle structure. J. Cell Biol. 116, 1071–1080 (1992) 1740466 10.1083/jcb.116.5.1071 PMC2289364

[b23] Roy A. K. *et al.* Regulation of androgen action. Vitam. Horm. 55, 309–352 (1999). 9949684 10.1016/s0083-6729(08)60938-3

[b24] Dehm S. M. & Tindall D. J. Molecular regulation of androgen action in prostate cancer. J. Cell Biochem. 99, 333–344, doi: 10.1002/jcb.20794 (2006). 16518832

[b25] Oettgen P. *et al.* PDEF, a novel prostate epithelium-specific ets transcription factor, interacts with the androgen receptor and activates prostate-specific antigen gene expression. J. Biol. Chem. 275, 1216–1225 (2000). 10625666 10.1074/jbc.275.2.1216

[b26] Sood A. K. *et al.* Expression characteristics of prostate-derived Ets factor support a role in breast and prostate cancer progression. Hum. Pathol. 38, 1628–1638, doi: 10.1016/j.humpath.2007.03.010 (2007). 17521701 PMC2121591

[b27] Sharrocks A. D. The ETS-domain transcription factor family. Nat. Rev. Mol. Cell Biol. 2, 827–837, doi: 10.1038/35099076 (2001). 11715049

[b28] Abbas T. & Dutta A. p21 in cancer: intricate networks and multiple activities. Nat. Rev. Cancer 9, 400–414, doi: 10.1038/nrc2657 (2009). 19440234 PMC2722839

[b29] Denard B. *et al.* The membrane-bound transcription factor CREB3L1 is activated in response to virus infection to inhibit proliferation of virus-infected cells. Cell Host Microbe 10, 65–74, doi: 10.1016/j.chom.2011.06.006 (2011). 21767813 PMC3139916

[b30] Jascur T. *et al.* Regulation of p21(WAF1/CIP1) stability by WISp39, a Hsp90 binding TPR protein. Mol. Cell, 17, 237–249, doi: 10.1016/j.molcel.2004.11.049 (2005) 15664193

[b31] Zhang L. *et al.* TRIM39 regulates cell cycle progression and DNA damage responses via stabilizing p21. Proc. Natl. Acad. Sci. USA 109, 20937–20942, doi: 10.1073/pnas.1214156110 (2012) 23213251 PMC3529087

[b32] O’Shea E. K., Rutkowski R. & Kim P. S. Evidence that the leucine zipper is a coiled coil. Science 243, 538–542 (1989). 2911757 10.1126/science.2911757

[b33] Karantanos T., Corn P. G. & Thompson T. C. Prostate cancer progression after androgen deprivation therapy: mechanisms of castrate resistance and novel therapeutic approaches. Oncogene 32, 5501–5511, doi: 10.1038/onc.2013.206 (2013). 23752182 PMC3908870

[b34] Heinlein C. A. & Chang C. Androgen receptor in prostate cancer. Endocr. Rev. 25, 276–308, doi: 10.1210/er.2002-0032 (2004). 15082523

[b35] Labrie C. *et al.* Androgen-regulated transcription factor AIbZIP in prostate cancer. J. Steroid Biochem. Mol. Biol. 108, 237–244, doi: 10.1016/j.jsbmb.2007.09.008 (2008). 17933519

[b36] Ghadersohi A. *et al.* Prostate-derived Ets transcription factor (PDEF) is a potential prognostic marker in patients with prostate cancer. Prostate 71, 1178–1188, doi: 10.1002/pros.21333 (2011). 21656828 PMC3112264

[b37] Johnson T. R. *et al.* Loss of PDEF, a prostate-derived Ets factor is associated with aggressive phenotype of prostate cancer: regulation of MMP 9 by PDEF. Mol. Cancer 9, 148, doi: 10.1186/1476-4598-9-148 (2010). 20550708 PMC2904725

[b38] Haller A. C. *et al.* High SPDEF may identify patients who will have a prolonged response to androgen deprivation therapy. Prostate 74, 509–519, doi: 10.1002/pros.22770 (2014). 24375440 PMC4410264

[b39] Sood A. K., Kim H. & Geradts J. PDEF in prostate cancer. Prostate 72, 592–596, doi: 10.1002/pros.21461 (2012). 21796651

[b40] Moussa O. *et al.* PDEF is a negative regulator of colon cancer cell growth and migration. J. Cell Biochem. 108, 1389–1398, doi: 10.1002/jcb.22371 (2009). 19830706 PMC3348703

[b41] Sabherwal Y. *et al.* PDEF downregulates stathmin expression in prostate cancer. Int. J. Oncol. 40, 1889–1899, doi: 10.3892/ijo.2012.1392 (2012). 22378487

[b42] Newman J. R. & Keating A. E. Comprehensive identification of human bZIP interactions with coiled-coil arrays. Science 300, 2097–2101, doi: 10.1126/science.1084648 (2003). 12805554

[b43] Cui M. *et al.* OASIS modulates hypoxia pathway activity to regulate bone angiogenesis. Sci. Rep. 5, 16455, doi: 10.1038/srep16455 (2015). 26558437 PMC4642342

[b44] Kanemoto S. *et al.* Luman is involved in osteoclastogenesis through the regulation of DC-STAMP expression, stability and localization. J. Cell Sci. 128, 4353–4365, doi: 10.1242/jcs.176057 (2015). 26503158 PMC4712816

[b45] Costes S. V. *et al.* Automatic and quantitative measurement of protein-protein colocalization in live cells. Biophys. J. 86, 3993–4003, doi: 10.1529/biophysj.103.038422 (2004). 15189895 PMC1304300

