# Supplementary material for: The androgen-induced protein AIbZIP facilitates proliferation of prostate cancer cells through downregulation of p21 expression
Source: Sci Rep. 2016 Nov 17;6:37310. doi: 10.1038/srep37310 (PMC5112536; doi:10.1038/srep37310)
Supplement: Supplementary Information [file srep37310-s1.pdf]

The androgen-induced protein AIbZIP facilitates proliferation of prostate cancer cells through downregulation of p21 expression

Xiang Cui, Min Cui, Rie Asada, Soshi Kanemoto, Atsushi Saito, Koji Matsuhisa, Masayuki Kaneko
& Kazunori Imaizumi

Supplementary Information

Supplementary Figure S1.

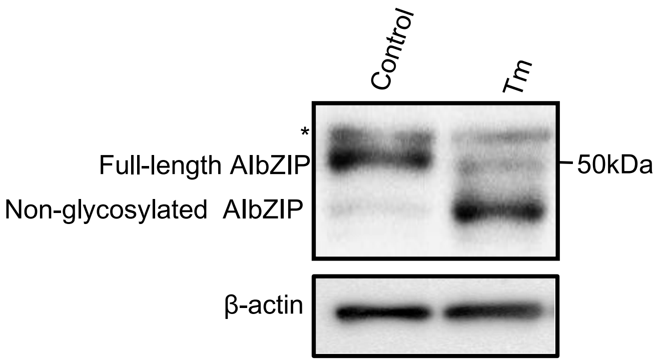

Supplementary Figure S1. WB analysis for AIbZIP in LNCaP cells treated with tunicamycin LNCaP cells were treated with 3 μg/ml tunicamycin (Tm). Asterisk: nonspecific bands. β-actin was used as a loading control.

Supplementary Figure S2.

-1K GGCAAAAAAATAAAAATAATCAATCAACCCTGAGTTTTGGTATTACTACTTTCTAGTCATGGATCTGCCTCCTCATAGG
TAAAATTTGCAAAATATTACCCGCCGTGTGTGCCTCAGAACTGTTATGGATTAAGGAAGAGAACAATTTTTATGAAAA
CGTTTTGTAAATTATACAAGTGTGGGACATAATGTTCCCGGTAACTGTATGCAAACAATAAGACAACAAACGCAAAAG
GCTTTTTAACTTGTTGCAGTTTTCCTGCCCCGTGTCCCTCCGTGAATGAGCAGCGTGCAGCGAGCGTGTGCGCACA
GCTGCTGTTCTCCATTAACGCCACACTGCTCGGCTTGCGGACGGGGAAACCCGCCATCGCGCTCCTGCCGCCTGC
CGAGCGAGGATCACCGCTCCACTGGGGTCAGCGGGCTGTTGACTCCCCGCCTGGGTGAAACCGGAGCTTCCGAGT
CACGTGGCGCGCGAAGACAAACCTGGCTGCTCAGGGTTTCCCGGAGCTTTCTCCCAGCTTCCCGGCGATTCATATT
CTGGCGCTTACCCCTCCCTTTAGGCTGGCGGAACCCAATCCCCGCCTGACAAATAAGTCCCGCCGGCCGGACGCA
GTGGCTCGCGCCTGTGGTCCTAGCTGCTCGCGGTGCTGAGGCGGGAGAATCACCTGAGCCCGGGAGGTCGGGGG
CTGCATGGAACCGTGATCGCACCACCGCACTCCAGCCTGGGCAACAGAGCGAGACCCTGTCTCCAAAAATAAGAAT
AAAGAAAAAAAAAAGAAAGAACTGCGGCCCCATTGGCCTGACTCTCCTGAACAGCCAAACTGTCTCCGACCAGTTAC
GGAAGAGGCGGCCTTGAGAGGGTAGTGTGCCATTGGCTCGATGTCCTGCCCCTCCGAAACCTAAGTCTTCAGCTTC
CAATCAGGACTCAGCTTTGGGAAGAGCGCCACGCGGTGGGCGAGGGGATGCCCTAGTAAATTCGCAGGTCTCTTG
ACTCTTTCCG

Supplementary Figure S2. The GGA(A/T) core sequences within the -1 kb upstream of *AIbZIP*
Sequences colored by green indicate the GGA(A/T) core sequences.

Supplementary Figure S3.

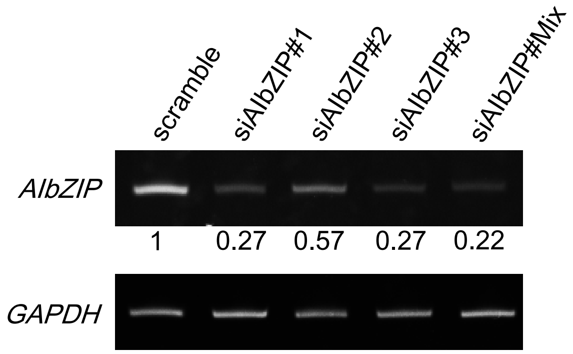

Supplementary Figure S3. The analysis for the expressions of *AIbZIP* in LNCaP cells transfected with siRNAs targeting *AIbZIP*

RT-PCR analysis for *AIbZIP* in LNCaP cells transfected with siRNAs targeting *AIbZIP* or scramble. Each siRNA (#1, #2, #3) for *AIbZIP* decreased the expression levels of *AIbZIP* to 27%, 57%, 27% respectively. A mixture of these siRNAs reduced the mRNA level of *AIbZIP* up to 78%. The numbers represent fold changes of mRNA levels. *GAPDH* was used as an internal control.

Supplementary Figure S4.

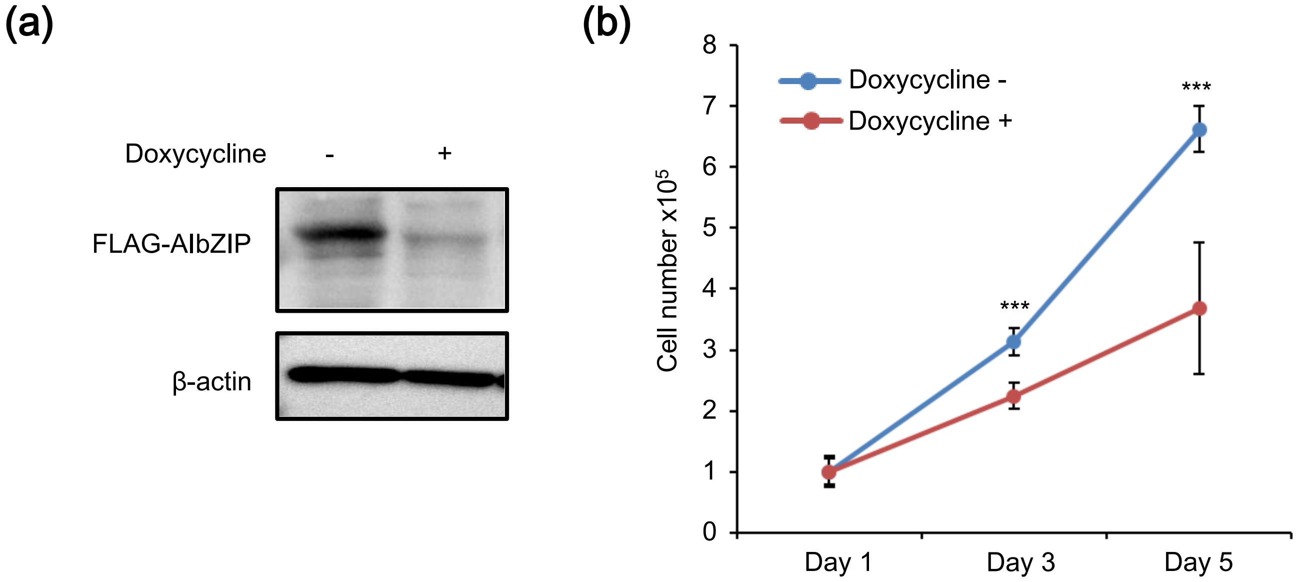

Supplementary Figure S4. Proliferation assay for LNCaP tet-off cells stably expressing FLAG-tagged AIbZIP

(a) WB analysis for FLAG-tagged AIbZIP in the LNCaP tet-off cells treated with doxycycline or not. (b) LNCaP tet-off cells were cultured with or without doxycycline, and the numbers of cells were counted at day 1, 3 and 5 (means ± s.d., n = 3; ***$P < 0.001$, vs. Doxycycline+).

Supplementary Figure S5.

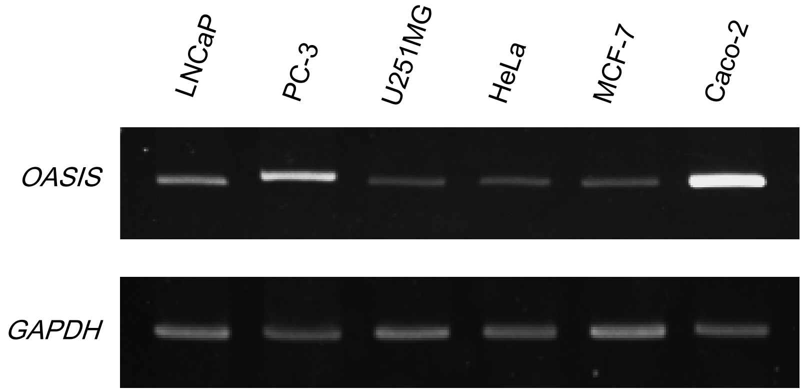

Supplementary Figure S5. RT-PCR analysis for *OASIS* in indicated cancer cell lines.

Supplementary Figure S6.

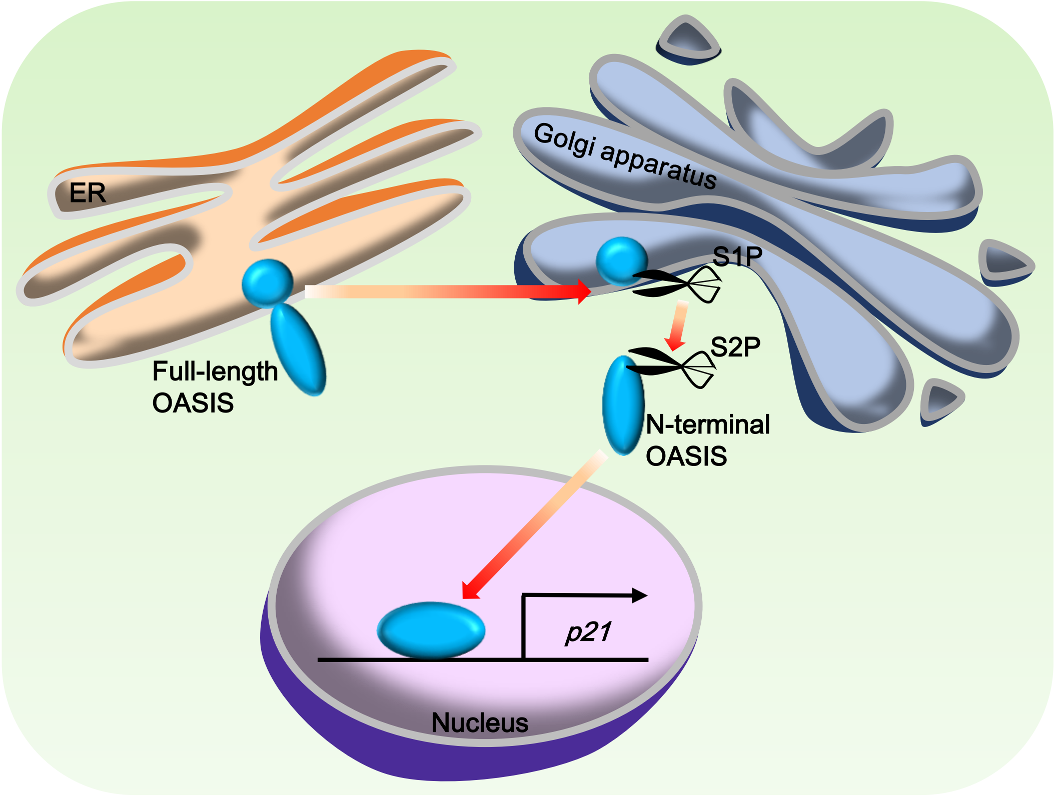

Supplementary Figure S6. Schematic representation for the mechanism of OASIS activation
In response to various stimuli, OASIS is translocated from the ER to the Golgi apparatus, and sequentially cleaved by site-1 protease (S1P) and site-2 protease (S2P). Cleaved N-terminal OASIS containing transcription activation and basic leucine zipper (bZIP) domains translocate to the nucleus to promote the transcription of target genes including *p21*.

Supplementary Figure S7.

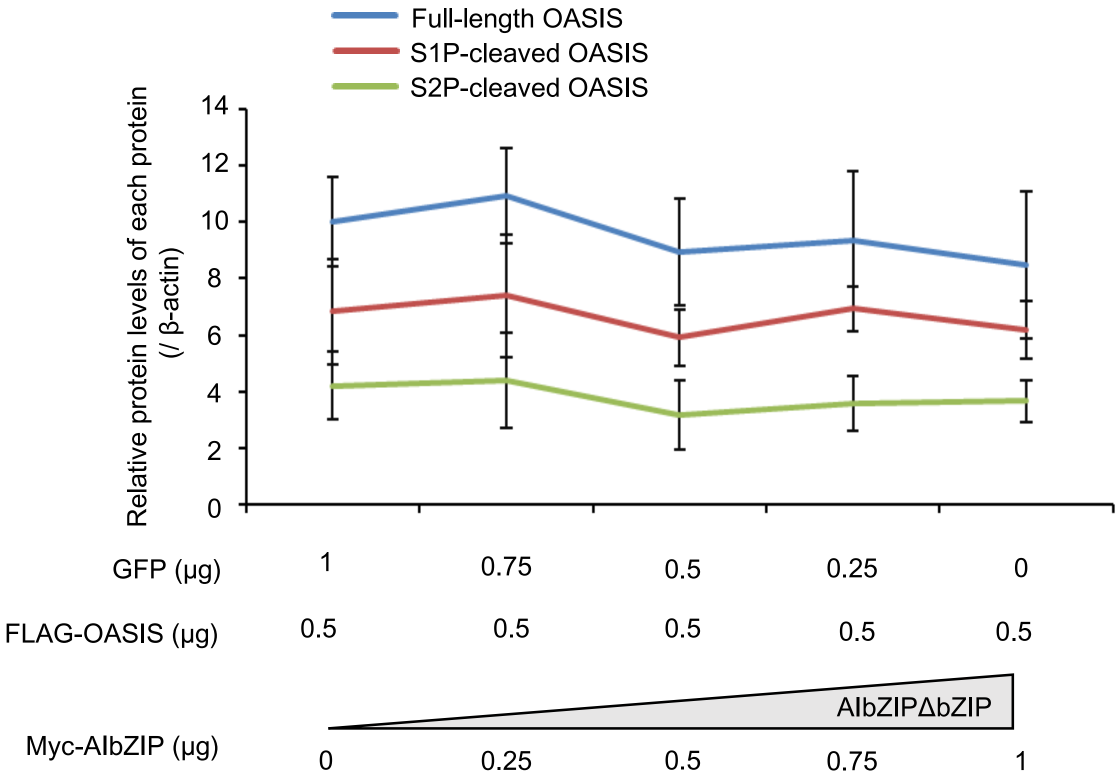

Supplementary Figure S7. Quantification for protein levels of full-length, S1P-, and S2P-cleaved OASIS in Figure 7(e). (means ± s.d., n = 3).

Supplementary Figure S8.

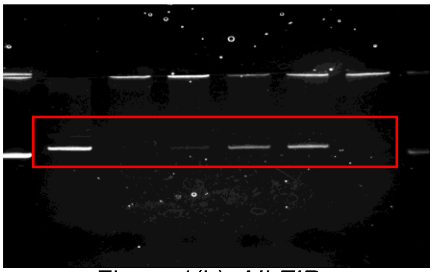

Figure 1(b) *AlbZIP*

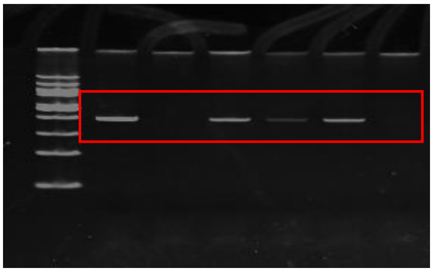

Figure 1(b) *AR*

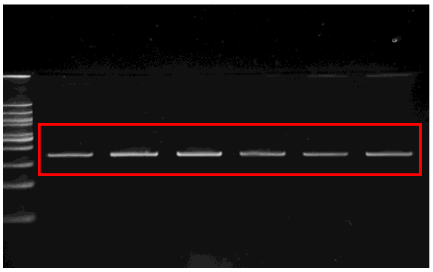

Figure 1(b) *GAPDH*

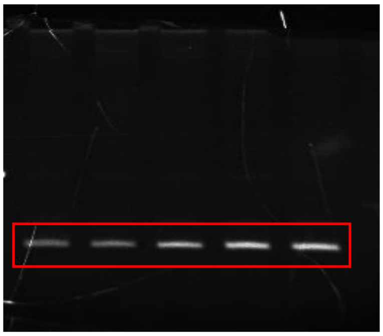

Figure 1(c) *AlbZIP*

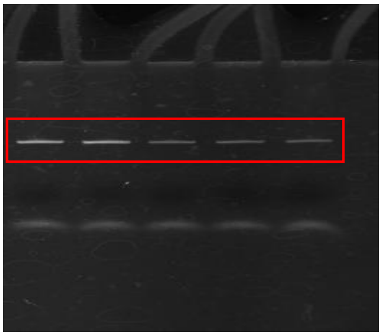

Figure 1(c) *GAPDH*

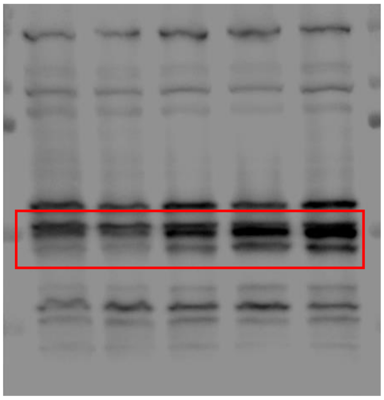

Figure 1(d) AlbZIP

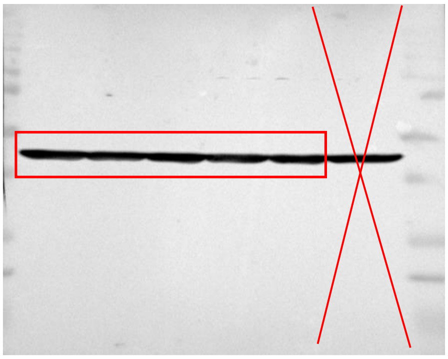

Figure 1(d) β-actin

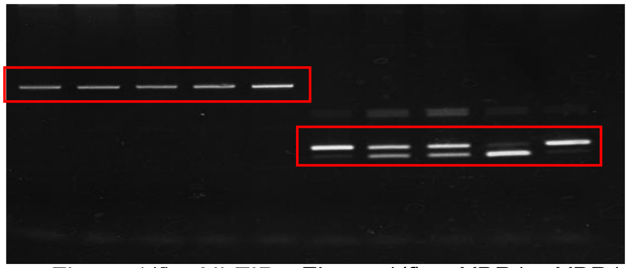

Figure 1(f) *AlbZIP* Figure 1(f) *uXBP1*, *sXBP1*

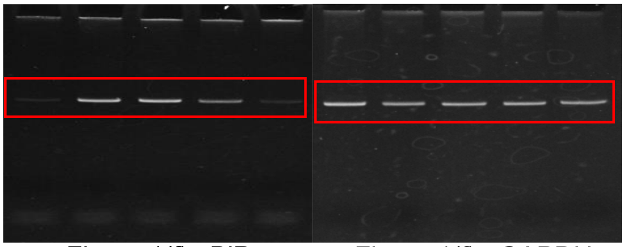

Figure 1(f) *BiP* Figure 1(f) *GAPDH*

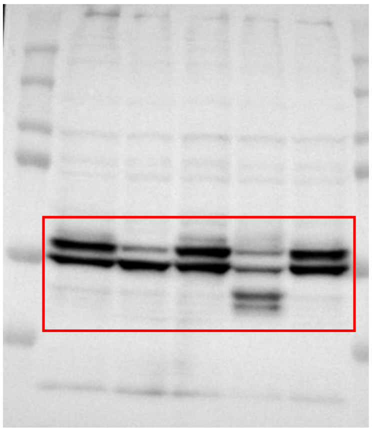

Figure 1(g) AlbZIP

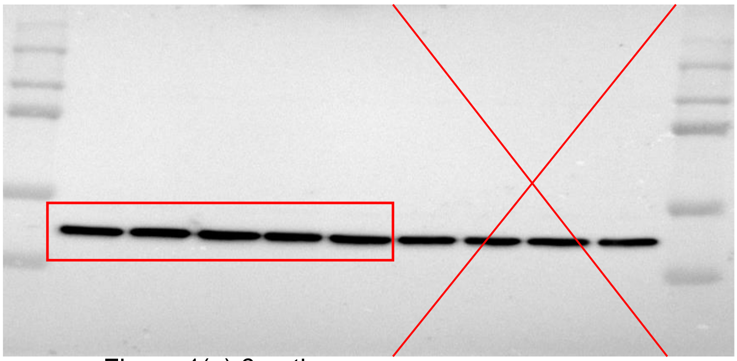

Figure 1(g) β-actin

Supplementary Figure S8. Full-length gels/blots of Figure 1(b), (c), (d), (f) and (g)

Supplementary Figure S9.

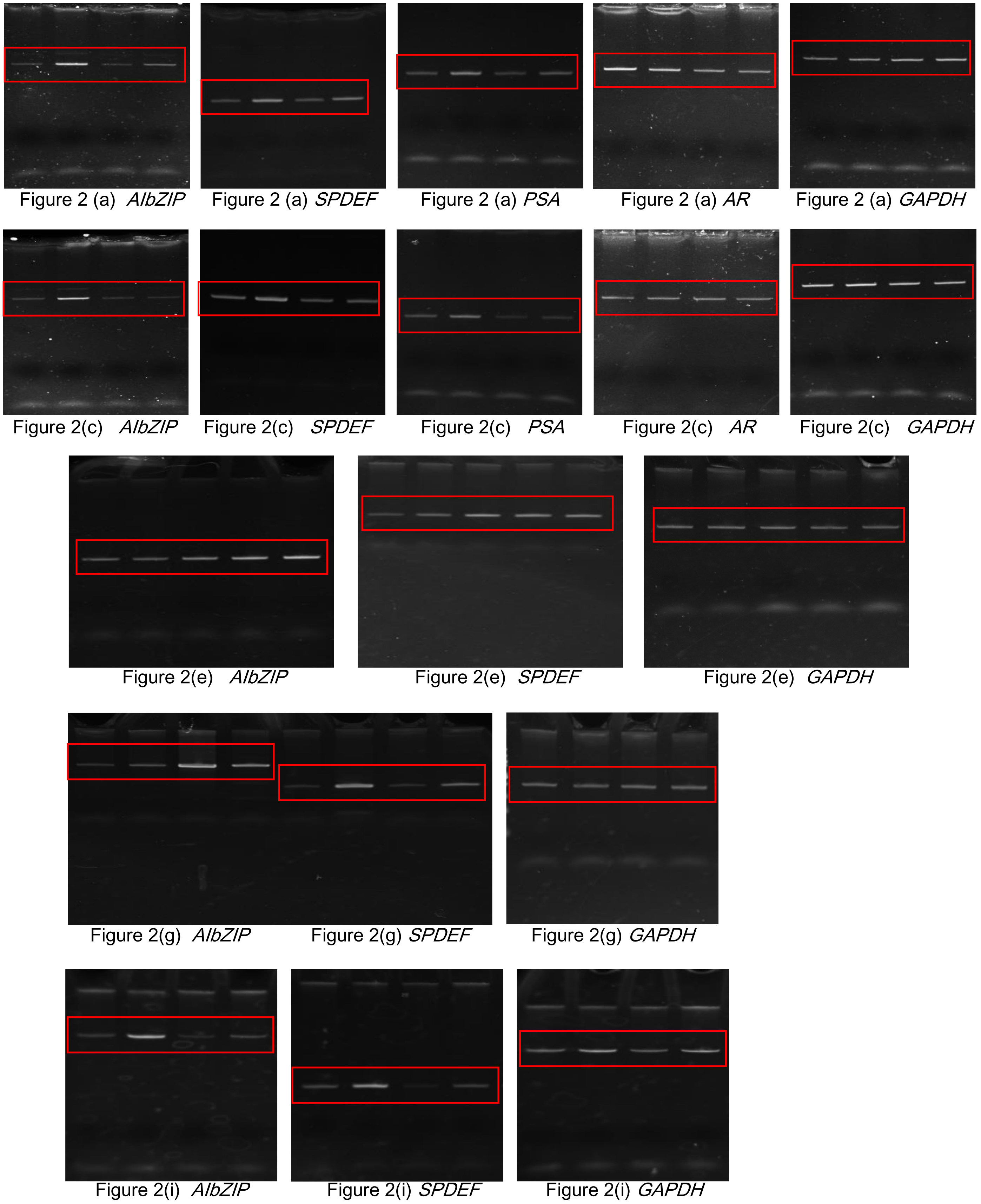

Supplementary Figure S9. Full-length gels of Figure 2(a), (c), (e), (g) and (i)

Supplementary Figure S10.

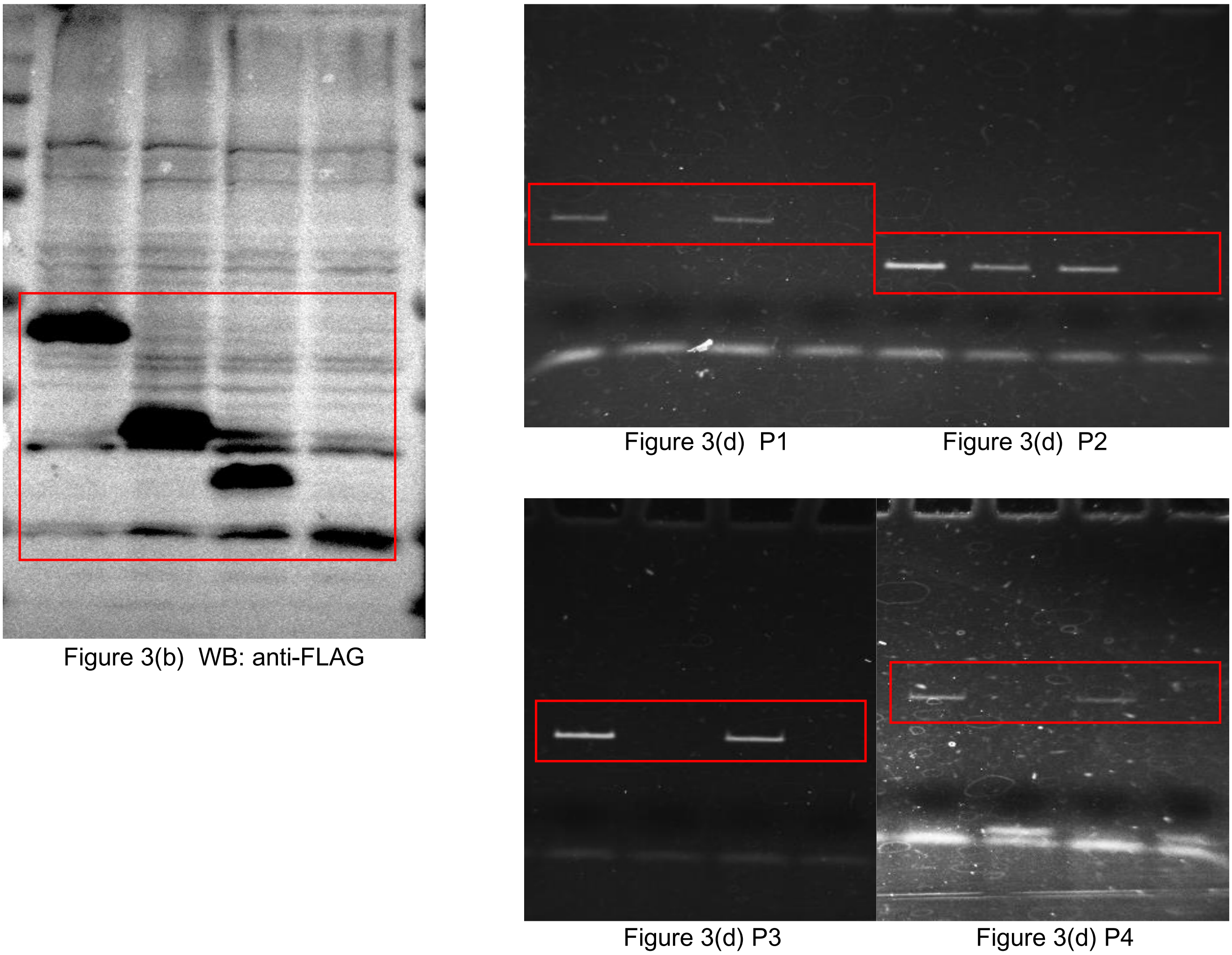

Supplementary Figure S10. Full-length gels/blots of Figure 3(b) and (d)

Supplementary Figure S11.

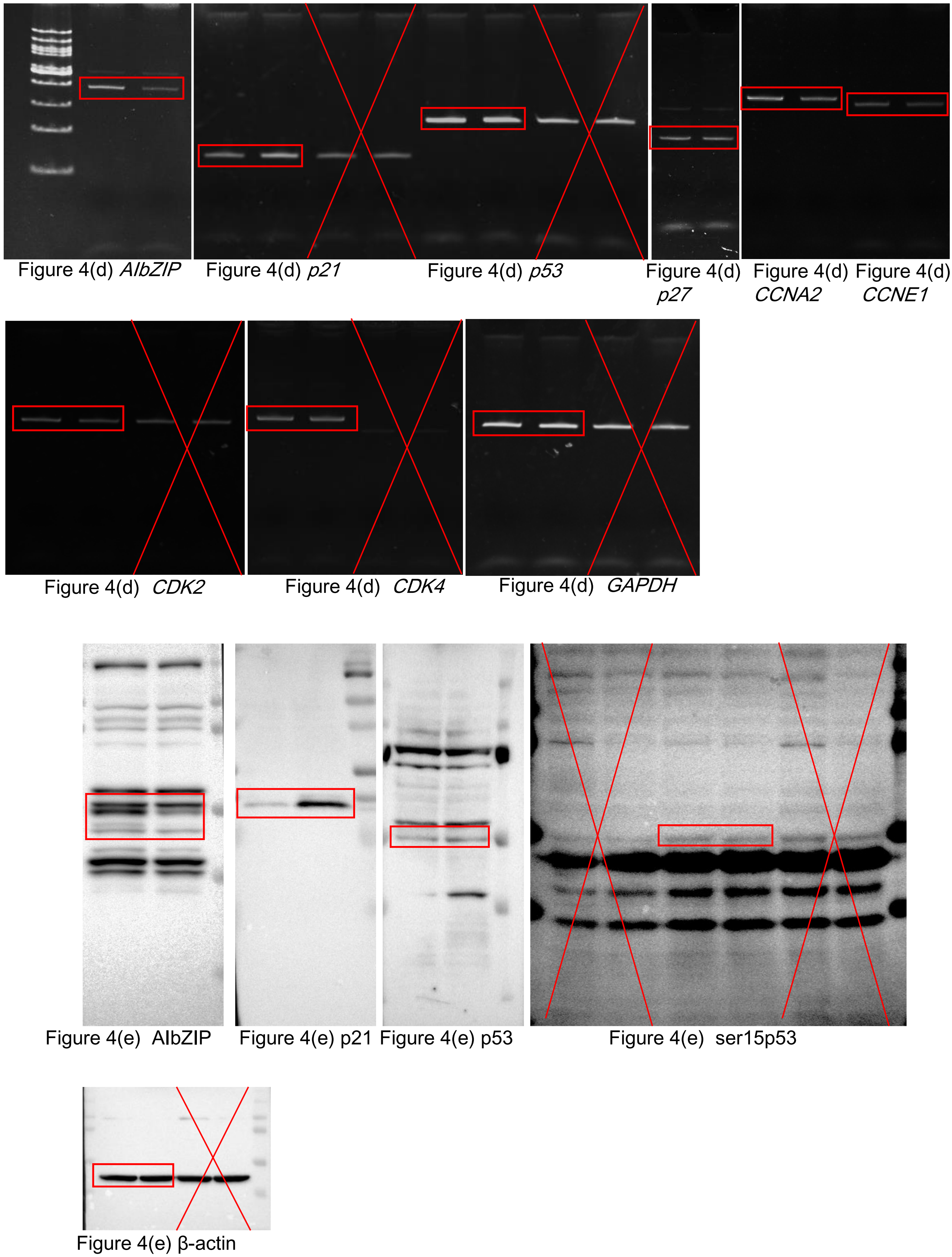

Supplementary Figure S11. Full-length gels/blots of Figure 4(d) and (e)

Supplementary Figure S12.

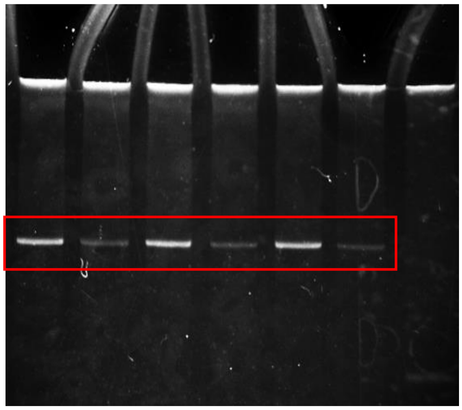

Figure 5(a) *AlbZIP*

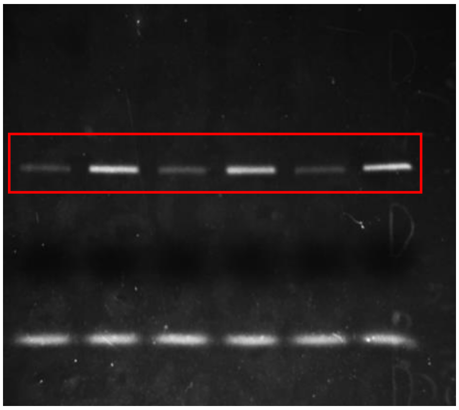

Figure 5(a) *p21*

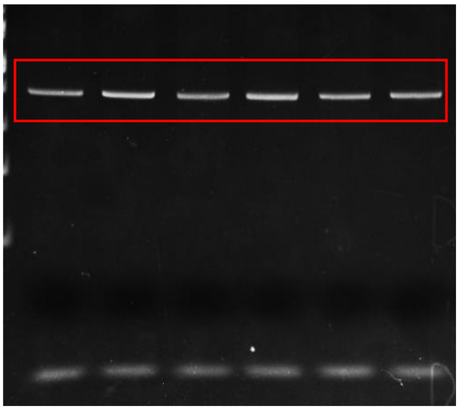

Figure 5(a) *GAPDH*

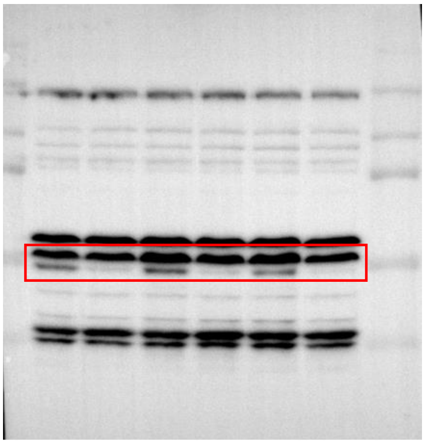

Figure 5(c) AlbZIP

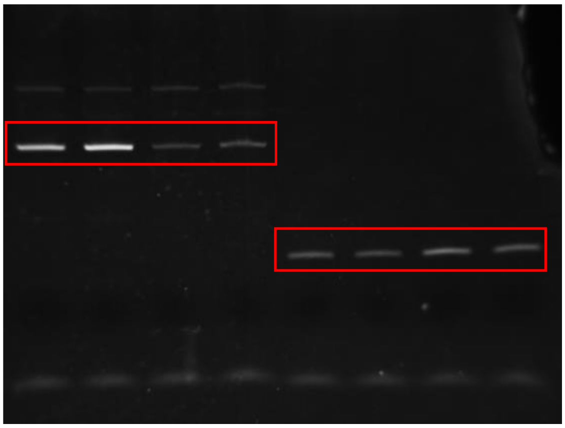

Figure 5(f) *AlbZIP* Figure 5(f) *p21*

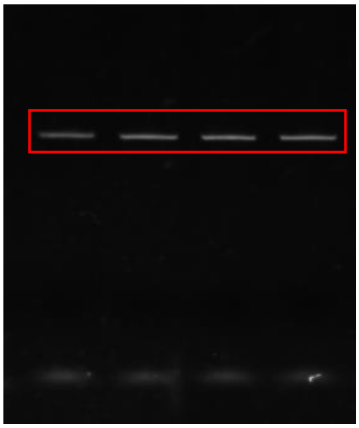

Figure 5(f) *GAPDH*

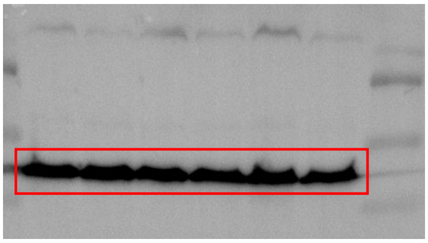

Figure 5(c) β-actin

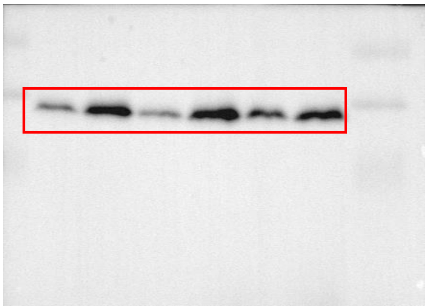

Figure 5(c) p21

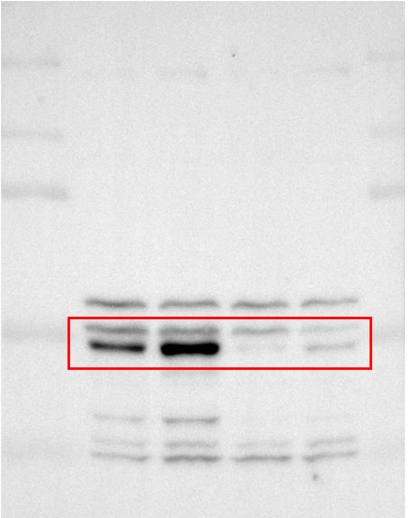

Figure 5(h) AlbZIP

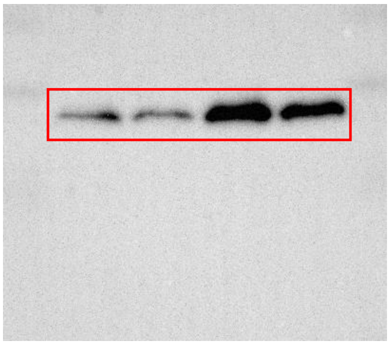

Figure 5(h) p21

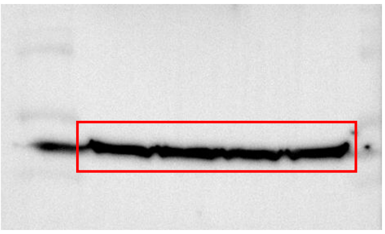

Figure 5(h) β-actin

Supplementary Figure S12. Full-length gels/blots of Figure 5(a), (c), (f) and (h)

Supplementary Figure S13.

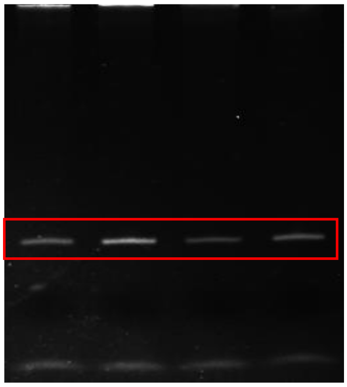

Figure 6(a) *p21*

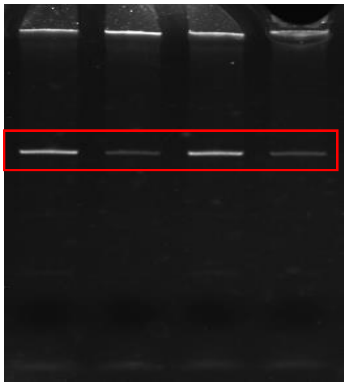

Figure 6(a) *AIbZIP*

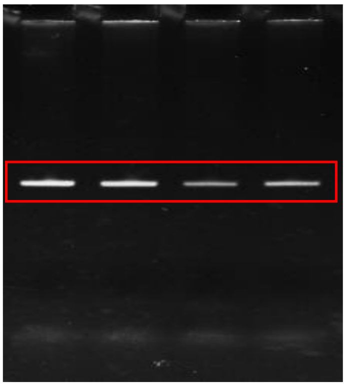

Figure 6(a) *OASIS*

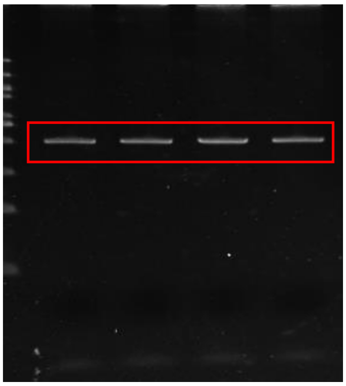

Figure 6(a) *GAPDH*

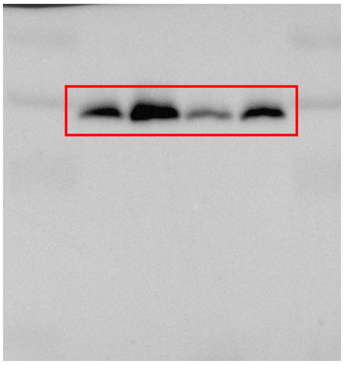

Figure 6(c) p21

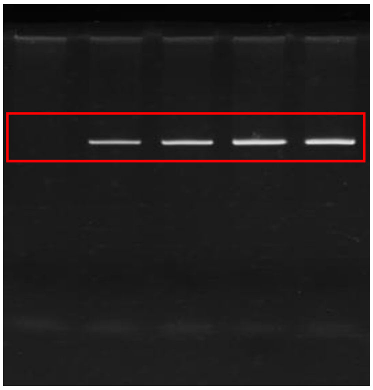

Figure 6(e) *AIbZIP*

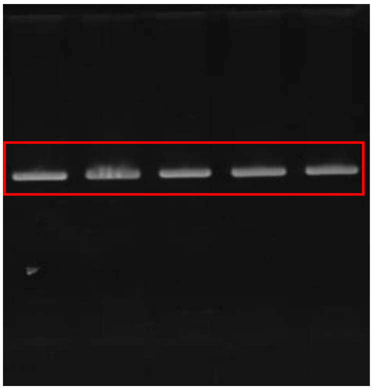

Figure 6(e) *OASIS*

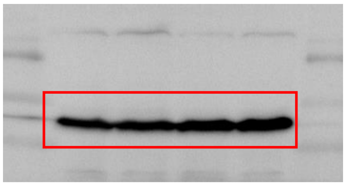

Figure 6(c) β-actin

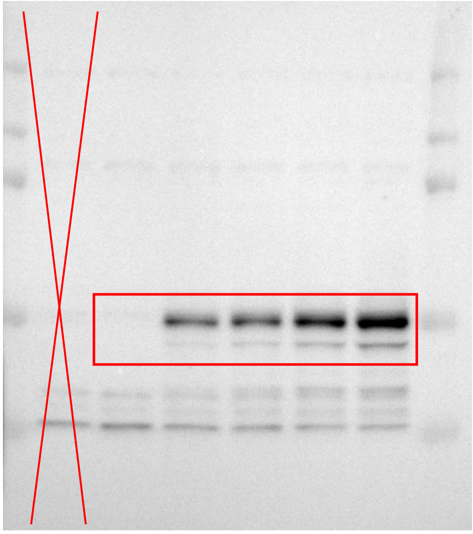

Figure 6(e) Myc-AIbZIP

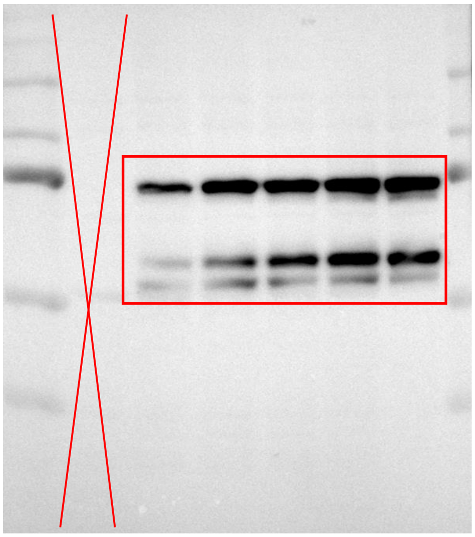

Figure 6(e) FLAG-OASIS

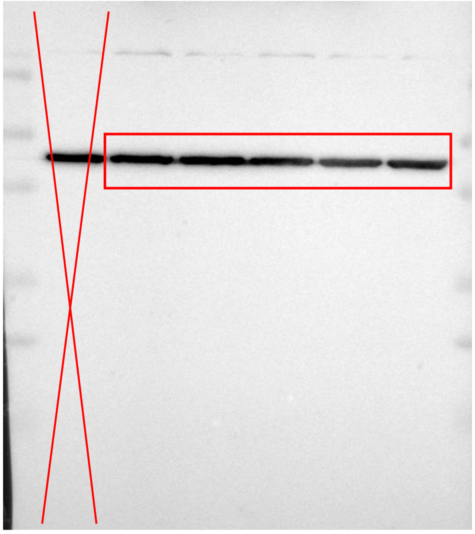

Figure 6(e) β-actin

Supplementary Figure S13. Full-length gels/blots of Figure 6(a), (c) and (e)

Supplementary Figure S14.

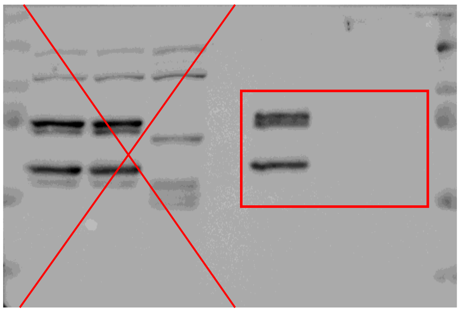

Figure 7(a) WB:OASIS
IP:AIbZIP

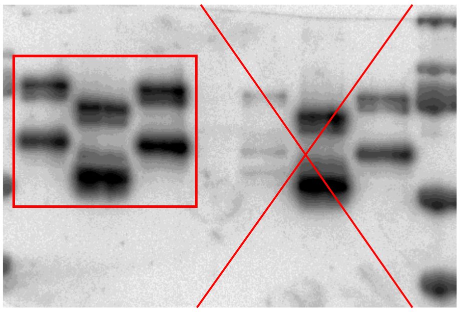

Figure 7(a) WB:OASIS
IP:OASIS

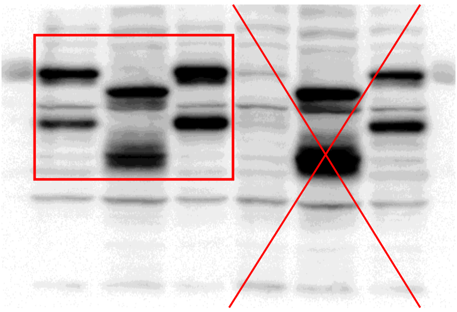

Figure 7(a) WB:OASIS
Input

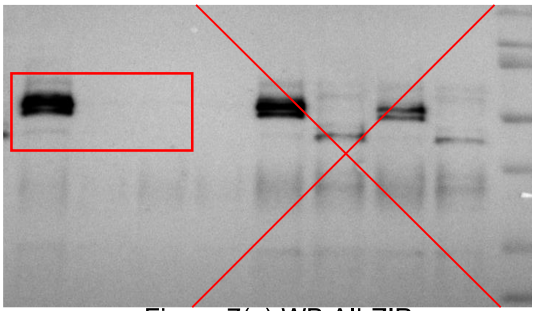

Figure 7(a) WB:AIbZIP
IP:OASIS

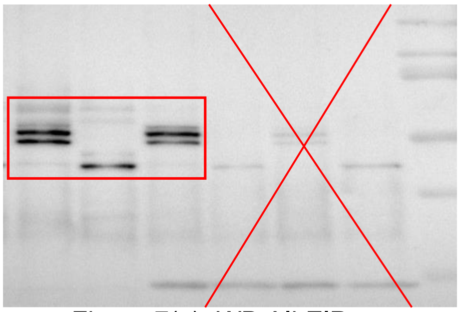

Figure 7(a) WB:AIbZIP
IP:AIbZIP

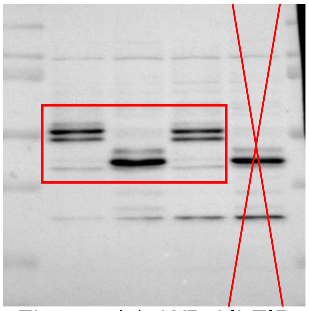

Figure 7(a) WB:AIbZIP
Input

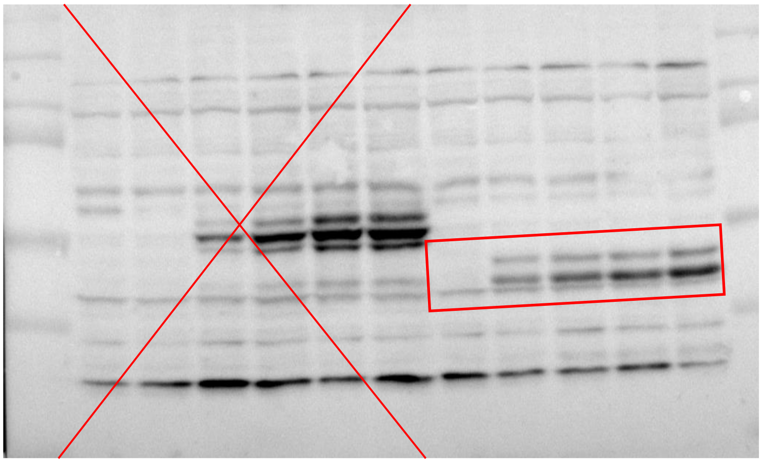

Figure 7(e) Myc-AIbZIP ΔbZIP

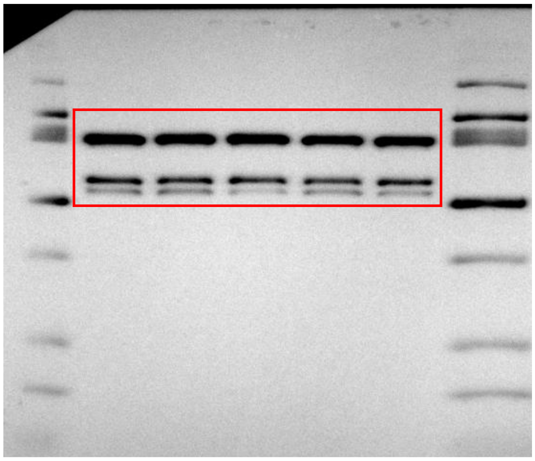

Figure 7(e) FLAG-OASIS

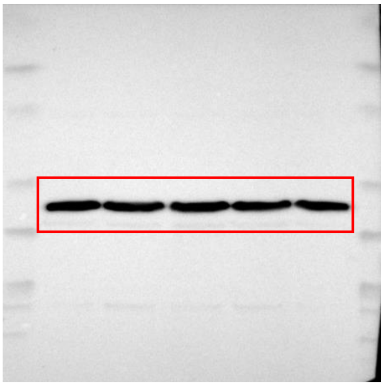

Figure 7(e) β-actin

Supplementary Figure S14. Full-length blots of Figure 7(a) and (e)

Supplementary Table 1.

List of primers used for plasmids construction		
**Gene**	**Primer**	**forward primer**
FLAG-AIbZIP	forward	5'-GGTACCATGGACTACAAGGATGACGATGACAAGGATCTCGGAATCCCTGAC-3'
	reverse	5'-TCACATCTCATCTGCATGCAG-3'
Myc-AIbZIP	forward	5'-ATGGAACAGAAACTGATCTCTGAAGAAGACATGACAAGGATCTCGGAATCCCTGAC-3'
	reverse	5'-TCACATCTCATCTGCATGCAG-3'
Myc-AIbZIPΔbZIP	forward	5'-TCCTCCAGACGCTAATTGCTCAAAC-3'
	reverse	5'-GTCTGGAGGACCCTCTCCTCTGCC-3'
FLAG-SPDEF	forward	5'-CCACCATGGACTACAAGGATGACGATGACAAGATGGGCAGCGCCAG-3'
	reverse	5'-TCAGATGGGGTGCACGAACTGGTAG -3'
FLAG-SPDEFΔETS	forward	5'-GCGGCCGCTCGAGTCTAGAGGGCCC-3'
	reverse	5'-TCACTGCCCGGAGCATGATGAGTCCACC-3'
FLAG-SPDEFΔSAM	forward	5'-GATGAAAGAGCGGACTTCACC-3'
	reverse	5'-GCCCACCACCATGGACTGCAC-3'
AIbZIP-5k	forward	5'-CAAGTGACTTATTCAGGGAGACTGC-3'
	reverse	5'-CGGAAAGAGTCAAGAGACCTG-3'
AIbZIP-5k to -1.7k	forward	5'-CAAGTGACTTATTCAGGGAGACTGC-3'
	reverse	5'-CCTTCCGACGAAACAGAGAAGTGATG-3'
AIbZIP-3k to -1.7k	forward	5'-CTGGGCAACAACAGCGAAAC-3'
	reverse	5'-CCTTCCGACGAAACAGAGAAGTGATG-3'
AIbZIP-1.7k	forward	5'-GGAAGGCTGCTTTGTCTCTTCACTAC-3'
	reverse	5'-CGGAAAGAGTCAAGAGACCTG-3'
AIbZIP-1.0k	forward	5'-CCTGGACCATCAGACATTAGAC-3'
	reverse	5'-CGGAAAGAGTCAAGAGACCTG-3'

Supplementary Table 2.

List of antibodies			
**antibody name**	**species**	**type**	**Manufacturer**
anti-β-actin	mouse	monoclonal	A2228, Sigma-Aldrich
anti-AIbZIP	mouse	monoclonal	H00148327-AP51, Abnova
anti-FLAG M2	mouse	monoclonal	F3165, Sigma-Aldrich
anti-FLAG	rabbit	polyclonal	600-401-383, ROCKLAND
anti-p21	mouse	monoclonal	SC-6246, Santa Cruz Biotechnology
anti-p53	mouse	monoclonal	#9282, Cell Signaling Technology
anti-phospho-p53 (Ser15)	rabbit	polyclonal	#9284, Cell Signaling Technology
anti-Myc	mouse	monoclonal	M192-3, MBL
anti-Myc	rabbit	polyclonal	562, MBL
anti-calnexin	mouse	monoclonal	ADI-SPA-860, Enzo
anti-GM130	rabbit	polyclonal	D6B1, Cell Signaling Technology
anti-Histone H3	mouse	monoclonal	SC-10809, Santa Cruz Biotechnology
anti-mouse IgG	mouse	monoclonal	G3A1, Cell Signaling Technology
anti-rabbit IgG	rabbit	polyclonal	Cell Signaling Technology

Supplementary Table 3.

List of primers used for RT-PCR		
Gene	forward primer	revers primer
AIbZIP	5'-GGTTCCGGTAACTAGGCT-3'	5'-AGACGCTTCTCCTCATCG-3'
PSA	5'-CCAGACACTCACAGCAAGGA-3'	5'-CTGAGGGTTGTCTGGAGGAC-3'
BiP	5'-GTTTGCTGAGGAAGACAAAAAGCTC-3'	5'-CACTTCCATAGAGTTTGCTGATAAT-3'
XBP1	5'-CAGCAGGTGCAGGCCCAGTTGTC-3'	5'-GACACTAATCAGCTGGGGAAAGAC-3'
AR	5'-TCCAAATCACCCCCCAGGAA-3'	5'-GACATCTGAAAGGGGGCATG-3'
SPDEF	5'-GTCAGCGGCCTGGATGAAAGA-3'	5'-AAGATGCCCTTCTCCTTGTTG-3'
p21	5'-GGAAGACCATGTGGACCTGT-3'	5'-GGCGTTTGGAGTGGTAGAAA-3'
p53	5'-AGTCACAGCACATGACGGAGG-3'	5'-TGGAGTCTTCCAGTGTGATGATG-3'
p27	5'-CAGCTTGCCCGAGTTCTACTACAG-3'	5'-AGGTCGCTTCCTCATCCCTG-3'
CCNE1	5'-CCAGGAAGAGGAAGGCAAACG-3'	5'-GTGTTGCTCAAGAAAGTGCTG-3'
CCNA2	5'-GCATGTCACCGTTCCTCCTTG-3'	5'-GTGATGTCTGGCTGTTTCTTC-3'
CDK2	5'-GGGCCTAGCTTTCTGCCATTC-3'	5'-GGAAACTTGGCTTGTAATCAGG-3'
CDK4	5'-CTTCCCATCAGCACAGTTCG-3'	5'-GGTGTAAGTGCCATCTGGTAG-3'
OASIS	5'-GAACATGGAGGACTTCTCCAATG-3'	5'-CGGGCTCTGCTCCTGCTTCAC-3'
GAPDH	5'-AGGTGAAGGTCGGAGTCAAC-3'	5'-GACGGTGCCATGGAATTTGC -3'

Supplementary Table 4.

List of siRNA sequences		
Gene	sense	anti-sense
scramble	5'-UUCUCCGAACGUGUCACGU-3'	5'-ACGUGACACGUUCGGAGAA-3'
AlbZIP #1	5'-GAACCAAGAAUUACAGAAA-3'	5'-UUUCUGUAAUUCUUGGUUC-3'
AlbZIP #2	5'-CAGAAAAUCUGGAGACCCA-3'	5'-UGGGUCUCCAGAUUUUCUG-3'
AlbZIP #3	5'-GGAAAUAAGUUUUGAGUGA-3'	5'-UCACUCAAAACUUAUUCC-3'
AR	5'-AAGAAGGCCAGUUGUAUGGAC-3'	5'-GUCCAUACAACUGGCCUUCUU-3'
SPDEF	5'-CCUUCUACCUCUCCUACUU-3'	5'-AAGUAGGAGAGGUAGAAGG-3'

Supplementary Table 5.

List of primers used for ChIP assay		
primer number	forward primer	revers primer
1	5'-CTGAGTTTTGGTATTACTACTTTC-3'	5'-CATACAGTTACCGGGAACAT-3'
2	5'-TGCTGTTCTCCATTAACGCCACAC-3'	5'-AGTCAACAGCCCGCTGACCC-3'
3	5'-TGGGGTCAGCGGGCTGTTGAC-3'	5'-GTCAGGCGGGGATTGGGTTC-3'
4	5'-ACCCAATCCCCGCCTGACAAATAAG-3'	5'-TGGCTGTTCAGGAGAGTCAGGCCAA-3'
